# Depression and HIV: a scoping review in search of neuroimmune biomarkers

**DOI:** 10.1093/braincomms/fcad231

**Published:** 2023-08-25

**Authors:** Arish Mudra Rakshasa-Loots

**Affiliations:** Edinburgh Neuroscience, School of Biomedical Sciences, The University of Edinburgh, Edinburgh EH8 9JZ, UK; Family Centre for Research with Ubuntu (FAMCRU), Tygerberg Hospital, Department of Paediatrics and Child Health, Stellenbosch University, Cape Town 7505, South Africa; Department of Global Health and Infection, Brighton and Sussex Medical School, University of Sussex, Brighton BN2 5BE, UK

**Keywords:** human immunodeficiency virus, major depressive disorder, neuroinflammation, immunometabolism, therapeutic targets

## Abstract

People with HIV are at increased risk for depression, though the neurobiological mechanisms underlying this are unclear. In the last decade, there has been a substantial rise in interest in the contribution of (neuro)inflammation to depression, coupled with rapid advancements in the resolution and sensitivity of biomarker assays such as Luminex, single molecular array and newly developed positron emission tomography radioligands. Numerous pre-clinical and clinical studies have recently leveraged these next-generation immunoassays to identify biomarkers that may be associated with HIV and depression (separately), though few studies have explored these biomarkers in co-occurring HIV and depression. Using a systematic search, we detected 33 publications involving a cumulative *N* = 10 590 participants which tested for associations between depressive symptoms and 55 biomarkers of inflammation and related processes in participants living with HIV. Formal meta-analyses were not possible as statistical reporting in the field was highly variable; future studies must fully report test statistics and effect size estimates. The majority of included studies were carried out in the United States, with samples that were primarily older and primarily men. Substantial further work is necessary to diversify the geographical, age, and sex distribution of samples in the field. This review finds that alterations in concentrations of certain biomarkers of neuroinflammation (interleukin-6, tumour necrosis factor-α, neopterin) may influence the association between HIV and depression. Equally, the chemokines monocyte chemoattractant protein-1 (MCP-1) and interleukin-8 (IL-8) or the metabolic index kynurenine:tryptophan (Kyn:Trp), which have been the focus of several studies, do not appear to be associated with depressive symptoms amongst people living with HIV, as all (MCP-1) or most (IL-8 and Kyn:Trp) available studies of these biomarkers reported non-significant associations. We propose a biomarker-driven hypothesis of the neuroimmunometabolic mechanisms that may precipitate the increased risk of depression among people with HIV. Chronically activated microglia, which trigger key neuroinflammatory cascades shown to be upregulated in people with HIV, may be the central link connecting HIV infection in the central nervous system with depressive symptoms. Findings from this review may inform research design in future studies of HIV-associated depression and enable concerted efforts towards biomarker discovery.

## Teaser

Inflammation may be a central link explaining the high risk for depression among people living with HIV. In a scoping review, Mudra Rakshasa–Loots highlights biomarkers of inflammation and related processes that are associated with depressive symptoms in people living with HIV and offers suggestions for future directions of inquiry.

## Graphical Abstract

**Graphical abstract:**
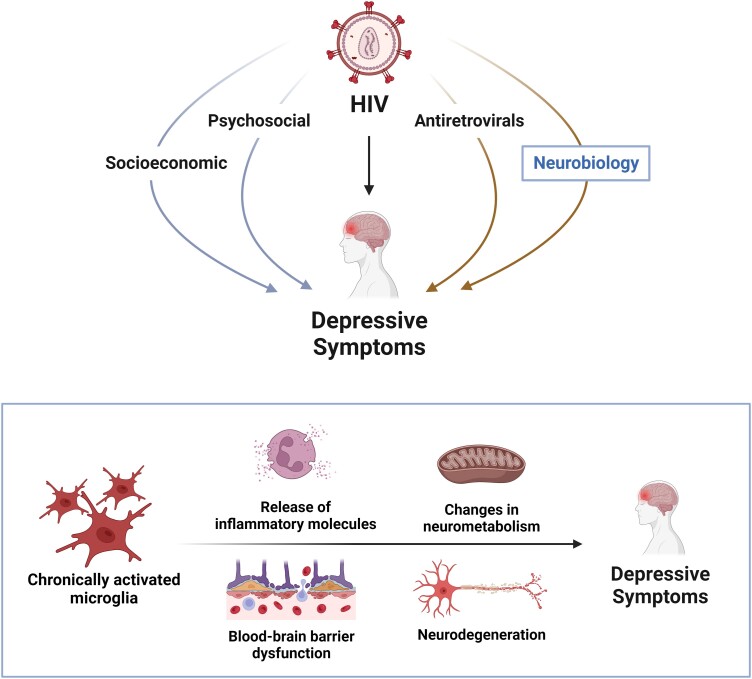

## Neuroinflammation in HIV-associated depression

‘No one cares about me, no one cares about my living or death. […] I cannot count on anyone, alone, lonely … Not counting how many days, it is in my mind all the time, always thinking about it, always thinking, ‘How do I live now?’ No housing, everyone spurns me, no money, unstable job, with illness, no one accepts me to work.’^1^

People with HIV experience depression at a rate at least twice as high as the general population.^2,3^ As the participant quoted above suggests, HIV status can impact an individual’s ability to access social support and employment. In addition, needing regular medical check-ups, adhering to drug regimens (with varied side-effects), and managing co-morbid conditions represent heavy personal and economic burdens for many people with HIV. Together with widespread stigma and marginalization, these factors contribute to the high risk for depression amongst people with HIV, especially for women with HIV and those in the Global South.^4,5^ However, as the brain is a key reservoir of HIV infection, it is likely that neurobiological processes also contribute to this high risk for depression. Identifying the precise mechanisms by which HIV leads to depression is crucial as it may enable us to identify individuals at greatest risk for developing depression, tailor the HIV care continuum, and deliver effective pharmacological or psychosocial interventions.

The last substantial review of the possible neurobiological mechanisms underlying HIV-associated depression was published by Del Guerra and colleagues in 2013.^6^ In this review, the authors hypothesized that HIV infection interacts with neurobiological and psychosocial factors to give rise to depression. Specifically, HIV infection in the brain results in sustained microglial activation, elevations in circulating cytokines, depletion of monoamines, and neurotoxicity (especially in dopaminergic neurons). Cerebral elevations in inflammatory neurometabolites and white matter abnormalities arising due to HIV infection persist despite viral suppression.^7^ These neurobiological consequences of central nervous system (CNS) infection may result in specific components of depression, such as cognitive loss (neuronal death), chronic fatigue (TNF-α elevations), and sickness behaviour (cytokine storms). HIV infection in the CNS may elicit a chronic neuroinflammatory response. Neuroinflammation is, in turn, associated with depressive symptoms in the general population. Thus, it is possible that neuroinflammation contributes to the increased risk for depressive symptoms among people living with HIV.^8^

In the intervening decade since the publication of Del Guerra *et al*.’s review, there has been continued interest in the contribution of (neuro)inflammation to depression, coupled with rapid advancements in the resolution and sensitivity of biomarker assays. Luminex, single molecular array, and similar next-generation multiplex assays have enabled the quantification of tens of biomarkers from single samples with enhanced sensitivity compared to enzyme-linked immunosorbent assays.^9,10^ These technologies have been leveraged in several recent investigations of biomarkers that may influence the association between HIV and depression.

To translate these findings into clinical practice, we must identify biomarkers that are reliably associated with depressive symptoms in people living with HIV. Depending on the study design and statistical methods, at least three questions may thus be investigated:

1. Is the onset of depressive symptoms in people living with HIV preceded by elevations in concentrations of certain biomarkers (*predictive biomarkers*);
2. Are depressive symptoms associated with certain biomarkers *only* in people living with HIV, and not in demographically comparable people without HIV, thus, helping identify an HIV-specific subtype of depression if one exists (*diagnostic biomarkers*); and
3. Is the severity of depressive symptoms in people living with HIV mediated by concentrations of certain biomarkers; that is, are these biomarkers at least partly mechanistically involved in the pathogenesis of symptoms and can therefore be targeted by antidepressant interventions (*therapeutic targets*).

By answering these questions, we may identify biomarkers that enable early risk stratification, phenotyping, and individualized interventions for depression in people living with HIV. Crucially, the utility of these biomarkers may extend beyond the context of HIV. Many inflammatory insults, including clinical conditions such as rheumatoid arthritis and psychosocial stressors such as early life adversity, result in an increased risk for depression.^11,12^ The connection between neuroinflammation and depression is therefore not unique to HIV. As a result, identifying biomarkers associated with HIV-associated depression may offer the 2-fold advantage of highlighting potential targets to reduce the prevalence of depression amongst people living with HIV (a community that remains globally under-served), and facilitating the translation of new and improved tools for stratification in the treatment of depression (a major global mental health concern).

The biomarkers tested in existing studies of HIV-associated depression are highly variable, in part due to the diversity and customizability of available biomarker panels. Recent reviews have narratively surveyed the literature on the potential role of inflammatory biomarkers in HIV-associated depression, but no attempt has been made to systematically capture and synergize these findings.^8,13^ This review aims to address this gap in the literature. Our objective was to synthesize recent evidence on associations between depressive symptoms and biomarkers of neuroinflammation or related biological processes in people living with HIV. Notably, in this scoping exercise, we did not seek to elucidate the mechanisms behind these associations but synthesize the reported associations themselves.

## Search methods

Using the Systematic Review Facility (https://syrf.org.uk/), we carried out a systematic search aimed at capturing all recent studies of people living with HIV which reported a measure of depression and at least one inflammatory biomarker. PubMed and Web of Science (Core Collection) were searched using detailed search terms, which are available in Supplementary Files 2–5. Studies eligible for inclusion in the review were those which: (i) were conducted in a human cohort including people with HIV, (ii) reported original data, (iii) were in English, (iv) were published between 1 January 2013 and 10 November 2022, (v) measured at least one biomarker of inflammation or related processes (as defined by a pre-determined list), and (vi) tested for an association between a biomarker of interest and depression or depressive symptoms. Studies were excluded if they involved participants with any other co-infections with HIV, such as tuberculosis or meningitis. Studies not reporting original data (such as reviews, meta-analyses, editorials, and case reports), animal studies, *in vitro* studies, and genetic studies were also excluded. The review protocol was not pre-registered. Study screening was carried out by a single reviewer.

In order to assess the clinical utility and strength of evidence for each inflammatory biomarker, available evidence from each included study was scored against the validation criteria in Table 1, modelled on criteria used by Oestreich and O’Sullivan.^14^ Data collection was carried out by a single reviewer. We first recorded whether each study reported a significant association between a biomarker of interest and depression or depressive symptoms (+1 score), which was our primary outcome of interest. Notably, we included studies that assessed depression as a clinical diagnosis (using, for instance, structured psychiatric interviews) and those that measured depressive symptoms using screening tools such as the Beck Depression Inventory. Diagnostic or screening tools used in each included study are listed in Supplementary Table 1.

**Table 1:** Criteria for validation of inflammatory biomarkers for HIV-associated depression

Grade	Definition	Rationale
0	No statistically significant differences in biomarker concentration between depressed and non-depressed people living with HIV	When assessed directly in a sample of people with HIV, suggests that the biomarker likely is not associated with depression in this population.
1	Biomarker concentration altered with depression amongst people living with HIV	Suggests a role for the biomarker in depression amongst people living with HIV, but whether this effect is HIV-specific cannot be inferred without direct comparisons to people without HIV.
2	Biomarker concentration altered with depression amongst people living with HIV but not among controls	Suggests strongly that the biomarker may be associated with an HIV-specific subtype of depression, thus, potentially a valid inflammatory biomarker of HIV-associated depression.
3	Biomarker concentration is associated with depressive symptom severity among people living with HIV but not amongst controls	As above; correlations with depressive symptom severity may suggest a dose response and thus, greater utility and sensitivity of the biomarker in community-based samples.

For studies reporting a significant association, we recorded the direction of association (positive/negative) and whether this association was significant only amongst participants living with HIV (+1 score). An HIV status-dependent association would be important for a truly useful predictive biomarker of HIV-associated depression. Although neuroinflammation is linked to depressive symptoms in the general population as well, it may be possible that certain inflammatory processes that are heightened during HIV infection (but not other immune challenges) may exacerbate the risk for depressive symptoms amongst people living with HIV. Biomarkers which are found to be associated with depressive symptoms in people living with HIV, but not in those *without* HIV, may thus represent an HIV-specific risk factor for depressive symptoms and thus at least partly explain the greater prevalence of depression in this population.

Finally, for studies that reported a significant association only amongst people living with HIV, we recorded whether there was a reported correlation between the biomarker concentration and a continuous scale of depressive symptom severity (+1 score). Correlations with depressive symptom severity may suggest a dose response and thus, indicate greater utility and sensitivity of the biomarker in community-based samples.

For all studies, we recorded all biomarkers assessed (including those which were reported only as part of a cluster of biomarkers), biofluids in which biomarkers were assessed, total number of participants (*N*), number of participants with HIV, mean [standard deviation (SD)] age of participants, % men in studies, recruitment country, % participants on ART, and % participants reported as virally suppressed. Where variables were reported as medians and interquartile ranges (IQR), means and SDs were imputed using equations (14) and (17) in Wan *et al*.^15^ Where variables were reported only for subgroups, mean (SD) for the full sample was imputed using equations in Table 6.5.a of the Cochrane Handbook.^16^ For biomarkers that were assessed in at least five publications, we extracted the type of screening or diagnostic tool used to measure depression or depressive symptoms, type of test statistic, and value of the effect size estimate. Data collection was carried out by a single reviewer.

## Search results and discussion

**Figure 1:**
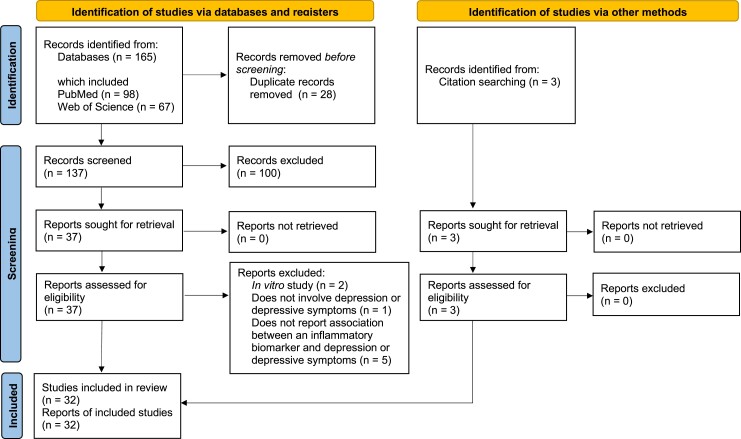
**PRISMA flowchart depicting the number of studies included in the review from database and manual citation searches.** Data from three unique studies was analysed and reported in two publications each; data from one unique study was analysed and reported in three publications.

**Figure 2:**
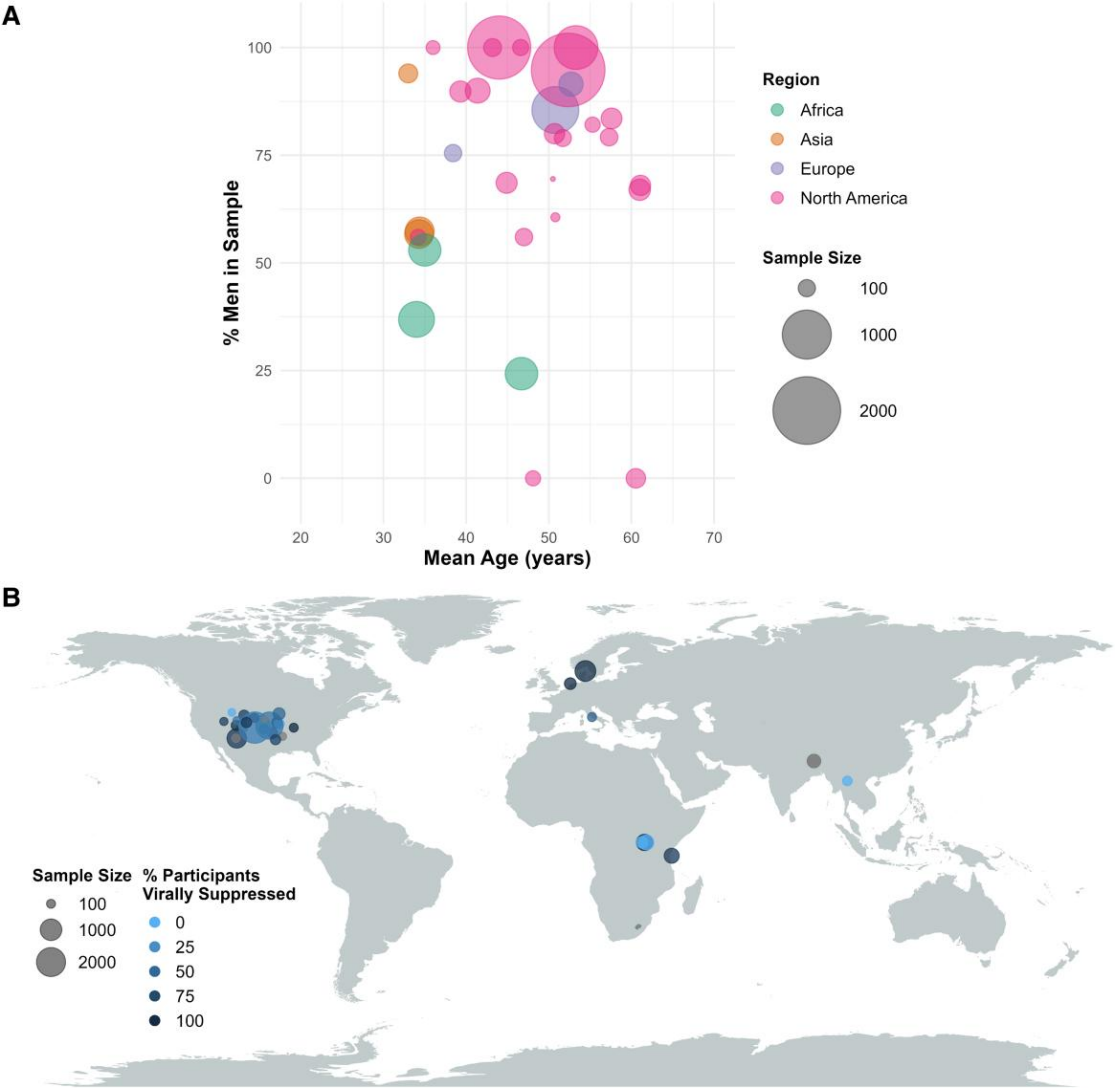
**Key characteristics of participant samples in included studies.** (**A**) Sex distribution (% men) and mean age of participants. Age of participants was not reported in two included studies. Colour of the bubbles corresponds to geographical region in which the study was conducted. (**B**) Global distribution of included studies and proportion of participants who were reported as virally suppressed. Each bubble represents one study, with size of the bubble proportional to the sample size of the corresponding study, and colour of the bubbles corresponding to the proportion of participants in the study who are reported as being virally suppressed. Bubbles are mapped to the country (not city) of recruitment, with position jitter for studies in the US to minimize overlapping data points.

Of 165 records identified from the two databases, three records identified from manual citation searching, and one record identified based on reviewer recommendation, a total of 33 studies (representing 28 unique samples and a cumulative 10 590 participants) were eligible for inclusion in this review (Fig. 1).^17–49^ Key characteristics of participants in these studies are summarised in Fig. 2. Overall, mean (SD) age of participants was 46.6 (8.8) years, with two studies not reporting the age of participants. Median (IQR) % of participants in the included studies who were men was 75.5% (56%, 90%). The overwhelming majority of studies (*n* = 23, 70%) recruited participants from the US. Five studies (two in the US, two in Uganda, and one in Thailand) involved participants who were not virally suppressed, whereas six studies did not report viral suppression status for their samples. At least a proportion of participants in all remaining studies were virally suppressed; median (IQR) % of virally suppressed participants overall was 79% (37%, 91%). Active HIV replication results in elevated inflammation, which may in turn worsen depressive symptoms.^50^ However, given that many participants in the included studies were on ART and virally suppressed, inferences about the impact of untreated HIV infection on inflammation and depression cannot be made.

**Figure 3:**
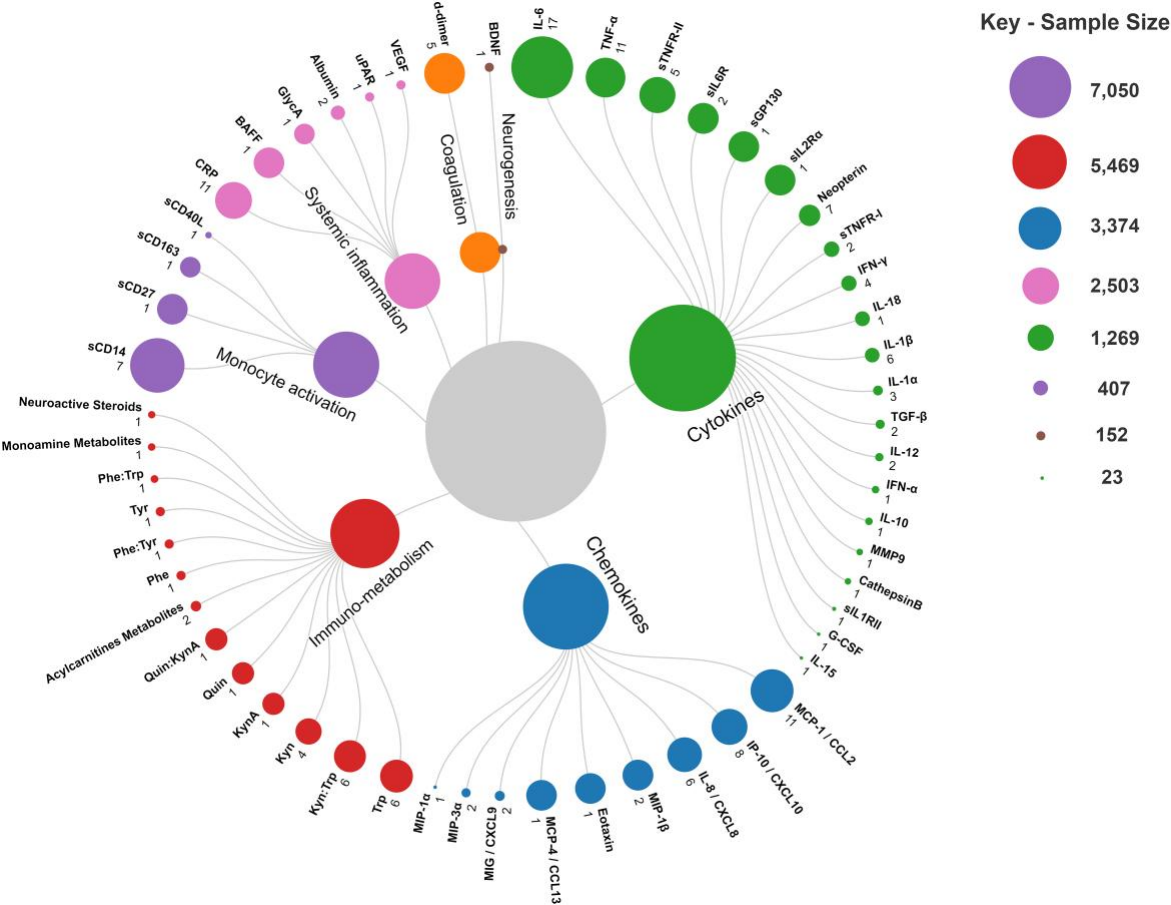
**Biomarkers of inflammation and related processes assessed in the included studies.** Included biomarkers were classified broadly into categories based on their primary putative function. Size of each circle is proportional to the cumulative sample size of publications involving that category or biomarker. Note that some publications leveraged data from overlapping samples. Each data label indicates the biomarker (in bold) and the number of publications in which the biomarker was assessed (in plain text). A list of abbreviations is available in Supplementary Table 5.

Collectively, the included studies tested associations of depression or depressive symptoms with 55 biomarkers of inflammation and related processes, of which IL-6, TNF-α, C-reactive protein (CRP) and MCP-1/CCL2 were assessed most frequently (Fig. 3). Biomarkers were assessed in a wide range of biofluids, including blood, cerebrospinal fluid, urine and saliva. A recent meta-analysis has revealed poor correlations between blood and cerebrospinal fluid (CSF) measures of inflammatory biomarkers,^51^ which suggests that care must be taken to choose a biofluid that is appropriate for the study design and with consideration for acceptability and scalability. Seven studies measured a single biomarker;^29,37,38,41,42,45,52^ all other included studies measured more than one biomarker. Three studies only reported associations involving a cluster or composite score of multiple biomarkers, such as effects for individual biomarkers could not be parsed out.^22,31,40^ Included biomarkers were broadly categorized based on their primary putative function in inflammation (or related processes) as follows: neuroinflammation, chemotaxis, systemic inflammation, monocyte activation, immunometabolism, coagulation, and neurogenesis. Note that biomarkers may be involved in multiple processes; for instance, many biomarkers that were categorized as neuroinflammatory in this review likely also play a role in systemic inflammation.

The majority of included studies used a screening tool to measure depressive symptoms, rather than a diagnostic interview to establish a clinical diagnosis of depression (Supplementary Table 1). Three studies reported non-specific associations between biomarkers and depressive symptoms: one as ‘mental health symptoms’,^33^ another as a composite of ‘depression or PTSD’,^41,52^ and another as an interaction of biomarkers with depression and neurocognition.

Formal meta-analyses were not possible in this review for a number of reasons. First, there was substantial heterogeneity in the scales used to measure depressive symptoms and the biofluids in which biomarkers were measured. Of 55 included biomarkers, 30 (54.5%) were assessed in only one publication, and a further 9 (16.4%) were assessed in two publications. We extracted effect size estimates for biomarkers which were assessed in at least five publications (Supplementary Table 1). Reporting of effect sizes in a substantial proportion of these studies was poor: of 115 reported associations, 29 (25.2%) did not include any test statistics or measure of effect size at all, whereas a further 10 (8.7%) were only reported collectively for a cluster of biomarkers, such that effect sizes for individual biomarkers could not be parsed out. Other associations were reported using estimates such as % feature importance (*n* = 6, 5.2%) or scaled intensity (*n* = 3, 2.6%) which could not reliably be converted to a meta-analysable statistic. Therefore, at least 41.7% of reported associations were not meta-analysable. Given these issues, an assessment of publication bias or a meta-analysis of the strength of the associations between biomarkers and depressive symptoms was not possible.

Included studies adopted varied approaches to accounting for confounding demographic or clinical variables. Some studies included demographically and clinically comparable controls,^18^ some adjusted for confounding variables statistically,^19,21^ while others combined both approaches.^24,44^ Where available, we extracted data for associations from statistical models adjusted for relevant covariates.^53^ Four included studies reported an active hepatitis C co-infection in at least a proportion of their participant samples,^17,18,40,49^ whereas one study specifically excluded participants with co-infection during recruitment.^36^ Active co-infections were not reported in the remaining studies. Several studies also reported psychosocial factors that may influence inflammation. In particular, a number of included studies focused on participants living with HIV who had experienced sexual violence in their lifetime.^20,26,46^ Jones *et al*.^29^ specifically attempted to assess depression as one component of ‘syndemic burden’, which was defined as incidence of depression, cigarette use, low education, or obesity. They found that syndemic burden was associated with increased viral load, but not inflammatory biomarkers, in women living with HIV. Overall, only a proportion of included studies reported measurement of and statistical adjustment for potential clinical or psychosocial confounders.

**Figure 4:**
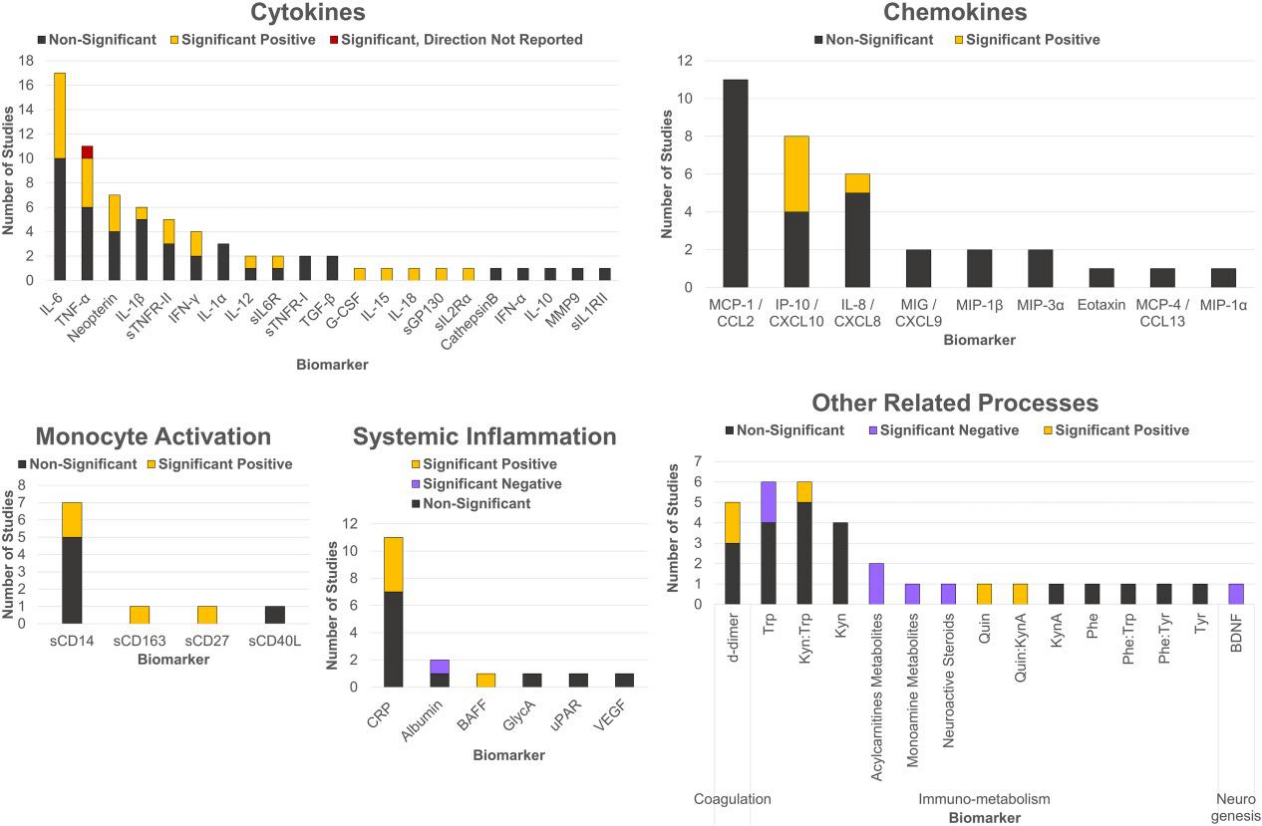
**Summary of findings from included studies on the association between inflammatory biomarkers and depression or depressive symptoms in people living with HIV.** Each column indicates the proportion of publications for that biomarker reporting a non-significant (in black), significant positive (in yellow), or significant negative (in purple) association. Note that some publications leveraged data from overlapping samples. A significant positive association implies that higher biomarker concentrations are linked to greater depression or depressive symptoms, whereas a significant negative association implies that lower biomarker concentrations are linked to greater depression or depressive symptoms. A list of abbreviations is available in Supplementary Table 5.

Proportion of studies reporting a significant association and direction of those associations are summarized in Fig. 4. Validation scores for biomarkers assessed in included studies are summarized in Table 2, and validation scoring for each included study is available in Supplementary Tables 2–4. The following sections report and discuss findings for broad categories of biomarkers across included studies.

**Table 2:** Summary of validation grading for included studies investigating associations between inflammatory biomarkers and depressive symptoms in people living with HIV

Category	Biomarker	Number of studies	Total validation grade	Maximum validation grade	Mean validation grade
Cytokines	IL-6	17	16	2	0.94
	TNF-α	12	12	2	1.00
	Neopterin	10	10	3	1.00
	IL-1β	6	3	1	0.50
	sTNFR-II	5	5	3	1.00
	IFN-γ	4	5	2	1.25
	IL-1α	3	1	1	0.33
	IL-12	2	3	2	1.50
	sIL6R	2	3	3	1.50
	sTNFR-I	2	0	0	0.00
	TGF-β	2	1	1	0.50
	CathepsinB	1	0	0	0.00
	G-CSF	1	2	2	2.00
	IFN-α	1	1	1	1.00
	IL-10	1	1	1	1.00
	IL-15	1	2	2	2.00
	IL-18	1	1	1	1.00
	MMP9	1	0	0	0.00
	sGP130	1	3	3	3.00
	sIL1RII	1	0	0	0.00
	sIL2Rα	1	3	3	3.00
Chemokines	MCP-1/CCL2	12	7	1	0.58
	IP-10/CXCL10	8	10	3	1.25
	IL-8/CXCL8	6	5	1	0.83
	MIG/CXCL9	2	1	1	0.50
	MIP-1β	2	1	1	0.50
	MIP-3α	2	1	1	0.50
	Eotaxin	1	1	1	1.00
	MCP-4/CCL13	1	1	1	1.00
	MIP-1α	1	0	0	0.00
Monocyte activation	sCD14	7	9	3	1.29
	sCD163	1	2	2	2.00
	sCD27	1	3	3	3.00
	sCD40L	1	0	0	0.00
Systemic inflammation	CRP	11	9	2	0.82
	Albumin	3	2	2	0.67
	B cell activating factor	1	3	3	3.00
	GlycA	1	1	1	1.00
	uPAR	1	1	1	1.00
	VEGF	1	1	1	1.00
Coagulation	d-dimer	5	6	3	1.20
immunometabolism	Kyn:Trp	7	5	2	0.71
	Trp	7	7	2	1.00
	Kyn	5	3	1	0.60
	Acylcarnitines Metabolites	2	4	3	2.00
	Phe	2	2	1	1.00
	Phe:Tyr	2	2	1	1.00
	Tyr	2	2	1	1.00
	KynA	1	0	0	0.00
	Monoamine Metabolites	1	3	3	3.00
	Neuroactive Steroids	1	1	1	1.00
	Phe:Trp	1	0	0	0.00
	Quin	1	1	1	1.00
	Quin:KynA	1	1	1	1.00
Neurogenesis	BDNF	1	3	3	3.00

## Cytokines

Cytokines are biomolecules produced by immune cells to activate, promote, or regulate the (neuro)inflammatory response. Concentrations of these biomarkers can be measured in biofluids including CSF, blood plasma or serum, saliva, or cervico-vaginal lavage. Included studies that assessed the relationship between neuroinflammatory cytokines and depressive symptoms are summarized in Supplementary Table 2.

The most frequently assessed neuroinflammatory cytokines in included studies were interleukin-6 (IL-6), tumour necrosis factor alpha (TNF-α), neopterin, interleukin-1 beta (IL-1β) and soluble tumor necrosis factor receptor-II (sTNFR-II). Numerous studies have found an association between circulating IL-6 or TNF-α concentrations and depressive symptoms in the general population, albeit with varied strengths.^54–61^ A recently published meta-analysis involving a cumulative 3075 participants also indicated that blood neopterin (regardless of type of blood sample) is significantly higher in participants with depression.^62^ The current review detected 17 publications that tested the relationship between IL-6 and depression or depressive symptoms, of which seven reported a significant positive association such that increased IL-6 was associated with greater risk for depression or depressive symptoms.^21,22,25,35,36,39^ Similarly, a proportion of studies exploring the relationship between TNF-α^21,22,35,39,48^ or neopterin^28,42,46^ and depressive symptoms observed significant positive associations. None of the studies reporting a significant association for IL-6 or TNF-α included participants without HIV. Saloner *et al*.^42^ observed a correlation between neopterin and depressive symptoms, which was significant only amongst people living with HIV, indicating that neopterin may be a valuable biomarker for HIV-associated depression. Together, these findings suggest that increased IL-6, TNF-α and neopterin may be associated with increased depression, including in people living with HIV. However, further studies that include appropriate controls are necessary to determine whether these cytokines (especially IL-6 and TNF-α) mediate the risk for depressive symptoms in a manner explicitly dependent on HIV status: that is, whether these associations are more pronounced in people living with HIV compared to those without HIV.

In line with findings related to TNF-α, two studies reported a significant association between increased serum concentrations of sTNFR-II and greater depressive symptoms,^31,33^ though Lu *et al*. only reported this association for sTNFR-II as part of a cluster of biomarkers [‘exploratory factor analysis-identified inflammatory processes’ (EIP)]. Similarly, Lu *et al*.^31^ also reported a significant association with depressive symptoms for soluble interleukin-2 receptor alpha (sIL2Rα), soluble interleukin-6 receptor (sIL6R) and soluble glycoprotein 130 (sGP130) but only as part of an EIP. Thus, no inferences can yet be drawn regarding the contributions of these individual biomarkers to HIV-associated depression.

Other neuroinflammatory cytokines for which a significant association with depressive symptoms was reported were interferon gamma (IFN-γ, two publications from an overlapping participant sample) and interleukin-12 (IL-12), interleukin-15 (IL-15) and interleukin-18 (IL-18), though these studies did not include participants without HIV and the findings from the overlapping sample in particular warrant further replication in independent samples.^21,22,33,49^ No significant associations with depression or depressive symptoms were observed in the included studies for the cytokines interleukin-1 alpha (IL-1α), soluble tumor necrosis factor receptor I (sTNFR-I), transforming growth factor beta (TGF-β), cathepsinB, interferon alpha (IFN-α), interleukin 10 or 12 (IL-10, IL-12), matrix metallopeptidase 9 (MMP9) or soluble interleukin-1 receptor II.

## Chemokines

Chemokines are biomolecules primarily involved in the recruitment of immune cells to locations of immunological challenges. Expression of these biomarkers is often regulated by neuroinflammatory cytokines such as TNF-α and IL-1β. Studies that assessed relationships between chemokines and depression in people living with HIV are summarized in Supplementary Table 3.

Monocyte chemoattractant protein-1 (MCP-1/CCL2) was the most frequently assessed chemokine in included studies. Of 11 studies, none reported a significant association between MCP-1 concentration and depression or depressive symptoms among people living with HIV. These findings are compatible with pre-clinical studies using a transgenic rat model of HIV, where Nemeth *et al*.^63^ found that animals who exhibited depression-like behaviour had increased concentration of MCP-1 in the hippocampus, but treatment with meloxicam did not rescue depression-like behaviour in these animals, despite successfully inhibiting *Mcp-1* gene expression. Therefore, although MCP-1 has been implicated in the risk for depression in the general population,^64,65^ the evidence suggests that this chemokine may not play a significant role in depression among people living with HIV specifically.

Interferon gamma-induced protein 10 (IP-10/CXCL10) and interleukin-8 (IL-8/CXCL8) are closely related chemokines belonging to the C-X-C family. Cassol *et al*.^18^ reported that increased plasma IP-10 was linked to depressive symptoms amongst people living with HIV but not controls. Three other studies also observed a significant positive association between IP-10 and depressive symptoms, but findings were limited only to men,^39^ in a small sample (*n* = 23),^49^ or reported only as part of a cluster of biomarkers.^66^ One study found that IL-8 concentrations were significantly higher amongst people with HIV and remitted depression compared to people with HIV and no depression,^39^ though several other studies with larger sample sizes did not find a significant association for IL-8. No notable associations were reported between depression or depressive symptoms in people living with HIV and the chemokines monokine induced by gamma interferon (MIG/CXCL9), macrophage inflammatory protein-1 alpha (MIP-1α/CCL3) macrophage inflammatory protein-3 alpha (MIP-3α/CCL20), macrophage inflammatory protein-1 beta (MIP-1β), eotaxin, or monocyte chemoattractant protein-4 (MCP-4/CCL13).

## Other inflammatory markers

Evidence for associations between depression or depressive symptoms in people living with HIV and biomarkers of systemic inflammation or monocyte activation are summarized in Supplementary Table 3.

### Systemic inflammation markers

C-reactive protein is an acute phase reactant which promotes phagocytosis and macrophage proliferation.^67^ During an inflammatory response, CRP is induced by inflammatory cytokines such as IL-6, TNF-α and IL-1β, which promote its production in the liver and secretion into plasma.^68^ CRP is elevated in people with HIV^69,70^ and people with depression,^54^ although the role of CRP in depression is unresolved due to contradictory findings. This review detected 11 studies which assessed CRP concentrations in people living with HIV, of which only four reported a significant association with depression or depressive symptoms.^33,37,39,41^ Notably, Rubin *et al*.^39^ only saw this association in women living with HIV and Saloner *et al*.^41^ only reported this association as an interaction between HIV, depressive symptoms, and neurocognition. None of these four studies reported that the association between CRP and depression was only significant for participants with HIV and not for those without HIV. Together with the existing contradictions in the literature on the role of CRP in depression, these findings suggest that CRP may not reliably influence HIV-associated depression.

Serum albumin was assessed in two studies, of which one study reported a negative association with depressive symptoms.^38^ The authors of this study speculate that albumin may be protective against depression, though such a claim would require substantial experimental work to verify. Furthermore, this study did not include participants without HIV and thus no conclusions can be drawn whether this effect may be influenced by HIV status. Other biomarkers of systemic inflammation assessed in the included studies were B cell activating factor (BAFF, for which Lu *et al*.^31^ reported a significant positive association with depressive symptoms, but only as part of a cluster of biomarkers), glycoprotein acetylation marker (GlycA), urokinase plasminogen activator surface receptor (uPAR/CD87), and vascular endothelial growth factor (VEGF), though no significant associations were reported for any except BAFF.

### Monocyte activation markers

Biomarkers of monocyte activation are expressed on the surfaces of monocytes and released in soluble form when these cells are activated. Neuroinflammatory cytokines such as IL-6 or IL-1β are involved in the induction of these markers.^71^ Included studies assessed four different biomarkers which are released by monocytes when they are activated: soluble cluster of differentiation 14, 163, 27, and 40 Ligand (sCD14, sCD163, sCD27 and sCD40L, respectively). Of the studies Investigating sCD14, Stewart *et al*.^44^ reported a significant correlation between somatic depressive symptoms and serum sCD14 concentrations amongst people living with HIV but not in people without HIV, which offers support for the validity of sCD14 as a biomarker for HIV-associated depression, though the difference in effect size for both groups was small. Similarly, Anderson *et al*.^17^ observed that higher plasma sCD163 was associated with increased odds for severe depressive symptoms only amongst people living with HIV. Lu *et al*.^66^ reported a significant association for sCD14 and sCD27 only as part of an EIP, whereas other studies captured in this review did not report a significant association between these biomarkers and depression. In all, sCD14 and sCD163 may drive the risk for depressive symptoms in people living with HIV, though further work is necessary to confirm these results.

### Astrocyte activation marker: glial fibrillary acidic protein

Glial fibrillary acidic protein (GFAP) is an intermediate filament protein which is constitutively expressed in astrocytes, and thus, serves as a marker of astrocyte activation and CNS pathologies.^72^ Ellis *et al*.^73^ recently investigated associations between CSF concentrations of GFAP with depressive symptoms in a sample of *n* = 212 people living with HIV in the US, with the majority (82.1%) of participants being men. They observed a statistically significant association between GFAP concentrations and total BDI-II scores with higher CSF GFAP correlating with higher BDI-II scores, suggesting that astrocytic activation may be associated with depressive symptoms in people living with HIV. These early findings indicate that GFAP may be a useful biomarker for depressive symptoms in this population. Further research into the function of astrocytes in neuroinflammation and depression in the context of HIV is warranted. Notably, this study was published in December 2022, outside the stated date range for our systematic searches, and was, therefore, not included in reported analyses in the rest of this review.

## Markers of related processes

Several included studies measured biomarkers of physiological processes that overlap with neuroinflammation. Findings from these studies are summarized in Supplementary Table 4.

### Immunometabolism markers

#### Kynurenine pathway metabolites

The kynurenine pathway of tryptophan metabolism sits at the intersection of neuroinflammation and metabolism. Typically, tryptophan (Trp) is catabolized by tryptophan hydroxylase into 5-hydroxytryptophan, the neurotransmitter otherwise known as serotonin and heavily implicated in the pathogenesis of depression.^74^ Under certain conditions, such as an inflammatory response, immune cells (including microglia and astrocytes) can instead metabolize tryptophan into kynurenine [Kyn, regulated by indoleamine 2,3-dioxygenase (IDO-1)], kynurenic acid (KynA) and quinolinic acid (Quin), all metabolites which form part of the kynurenine pathway.^75,76^ The ratio of kynurenine to tryptophan (Kyn:Trp) is an index of IDO-1 activity, which is triggered by key neuroinflammatory cytokines such as IL-6, TNF-α, IL-1β and IFN-α.^77,78^

Activation of the kynurenine pathway depletes available tryptophan for serotonin production and thus is considered a possible mechanism by which immune responses interact with neurotransmitter dysfunction to give rise to depressive symptoms. The kynurenine pathway has particular clinical relevance to HIV infection, which has been reviewed elsewhere.^79^ HIV viral proteins can activate the kynurenine pathway, in addition to inducing the release of pro-inflammatory cytokines which further promote this activation, leading to a feedforward loop of sustained tryptophan depletion.^80,81^

Several included studies tested for associations between Kyn or KynA and depressive symptoms, with none reporting a significant association. Five studies investigated associations between Trp in plasma or CSF with depressive symptoms. Of these, Martinez *et al*.^32^ and Vadaq *et al*.^45^ both observed that lower plasma tryptophan was significantly associated with greater depressive symptoms, though their samples did not include people without HIV. Finally, although the Kyn:Trp ratio was assessed in several studies, only Martinez *et al*.^32^ observed a statistically significant association between higher plasma Kyn:Trp ratio and greater depressive symptoms in people living with HIV, and the effect size of this association was small (+0.01 HSCL-D score for each 10 nM/µM increase in Kyn:Trp ratio). Conversely, Keegan *et al*.^30^ saw a trend towards lower plasma Kyn:Trp weakly associating with depression in aviremic people with HIV (compared to viremic people with HIV or people without HIV), though this difference was not statistically significant after Bonferroni correction. Taken together, these findings suggest that lower plasma Trp may be a useful biomarker of the risk for depression in HIV, but further work is necessary to resolve contradictory findings on the association between Kyn:Trp ratio and depression.

Quinolinic acid is considered a neurotoxic metabolite, leading to the hypothesis that increased production of Quin may be a driver of an inflammatory subtype of depression.^77^ Absolute concentrations of Quin are increased in untreated depression and correlate with depression severity.^82^ Furthermore, antidepressant behavioural activation therapy leading to reduction in depressive symptoms is significantly associated with decreased serum Quin.^83^ Crucially, this relationship has also been observed in the specific context of HIV. Drivsholm *et al*.^23^ saw that higher Quin:KynA ratio (but not absolute KynA concentrations) and higher absolute concentrations of Quin were both significantly associated with depression in a large sample of people with HIV. Given these findings, Quin may be a reliable biomarker of depression in people living with HIV.

#### Other neuroimmunometabolites

Studies included in this review did not find significant associations of tyrosine (Tyr), phenylalanine (Phe), Phe:Tyr ratio, or Phe:Trp ratio with depressive symptoms.^19,30^ Two publications from an overlapping participant sample^18,34^ found that monoamine metabolites (phenylacetate and 4-hydroxyphenylacetate), acylcarnitine metabolites (isovalerylcarnitine, isobutyrylcarnitine, and propionylcarnitine), and neuroactive steroids (pregnenolone sulphate, pregnanediol-3-glucuronide, and DHEA sulphate) were decreased in people living with HIV who experience depression. These findings suggest that these metabolites may be protective against depressive symptoms, though replication of these results in independent samples is warranted. Notably, Cassol *et al*.^18^ observed the effect of monoamines and acylcarnitines amongst participants *without* HIV as well, suggesting that these relationships are not dependent on HIV status.

### Coagulation marker: D-dimer

D-dimer is a biomarker of coagulation which is also elevated in systemic inflammation.^84^ Increased d-dimer concentration is independently linked to serious HIV-related conditions such as cardiovascular disease or immune reconstitution inflammatory syndrome (IRIS) and with depressive symptoms.^85–87^ The current review detected five studies that tested for associations between d-dimer and depressive symptoms, of which two reported a significant association.^25,44^ Notably, Stewart *et al*.^44^ observed that this association was only significant for people with HIV (and not for those without HIV), and that serum concentration of d-dimer was positively correlated with somatic symptoms of depression. Each SD increase in PHQ-9 somatic score was associated with a 6% increase in d-dimer concentration in people living with HIV, but no increase in people without HIV. These studies offer early evidence that d-dimer may be associated with depressive symptoms in a manner dependent on HIV status.

### Neurogenesis marker: brain-derived neurotrophic factor

Brain-derived neurotrophic factor (BDNF) is a regulator of neurogenesis and synaptic plasticity, with additional functions in apoptosis and T cell survival.^88^ BDNF binds to the receptor tropomycin receptor kinase B (TrkB) to promote neuronal and synaptic growth and survival. Given this, reduction in BDNF is associated with neuronal death and synaptodendritic injury. Decreased BDNF has been linked with depression in rigorous meta-analyses, though the effect size of this association is relatively small.^89^

BDNF is not a marker of neuroinflammation, but it does interact with HIV-induced neuroinflammatory processes in ways which are relevant to the current discussion. Notably, HIV infection in the brain leads to production of neuroinflammatory cytokines such as TNF-α and IL-1β, which have recently been shown to inhibit expression (TNF-α), retrograde transport (IL-1β) and anterograde transport (TNF-α) of BDNF.^90^ Concentrations of BDNF are elevated in CSF following CNS infections, suggesting that neuroinflammation influences BDNF expression.^91,92^ Furthermore, there is some evidence that BDNF signalling may exhibit cross-talk with the kynurenine pathway and associated immuno-metabolic dysfunctions.^93^ Pre-clinical studies show that induction of depression-like behaviours in rats was accompanied by decreased BDNF expression and increased IDO-1, Kyn, and Trp production, whereas inhibition of the kynurenine pathway significantly increased cortical BDNF levels.^94,95^ Therefore, BDNF signalling is influenced by neuroimmunometabolic responses known to be induced by HIV infection.

Given this evidence, it is possible that BDNF may be associated with the risk for depression in HIV. Woods *et al*.^47^ recently explored this question in a sample of older adults with and without HIV. In this study, the authors found that lower plasma BDNF concentrations were associated with higher scores on specific dimensions of mood disorders, notably Depression-Dejection (as measured by the Profile of Mood States, POMS). This association was only significant for participants with HIV, crucially demonstrating that the effect of BDNF on depressive symptoms may be dependent on HIV status. This study offers promising (though early) evidence that BDNF may be a useful biomarker of HIV-associated depression, with further work necessary to replicate this finding in diverse cohorts.

## Insights in absence

As the resident immune cells in the CNS, microglia are essential to the neuroinflammatory response. Under homeostatic conditions, microglia are highly ramified, whereas ‘activated’ microglia reacting to immunological challenges are amoeboid. These activated microglia—and, to some extent, astrocytes—perform key immune functions in the brain, such as phagocytosing cellular debris and triggering immunological cascades.^96^ For this reason, glial cell activation is widely considered to be the key characteristic of neuroinflammation. However, measuring glial cell activation directly in humans is challenging, as this requires access to brain tissue via biopsies or autopsies.

Positron emission tomography (PET) and magnetic resonance spectroscopy (MRS) allow for non-invasive *in vivo* assessment of certain biomarkers which are considered indirect measures of glial cell activation, namely, 18 kDa translocator protein, myo-inositol and choline. However, despite explicitly including search terms for TSPO, myo-inositol and choline, our systematic search did not detect any studies published since 2013 which examined changes in these neuroimaging biomarkers directly in relation to depression or depressive symptoms among people living with HIV. Newly developed radiotracers for PET ligands offer greater capacity to measure microglial activation *in vivo*.^97^ The leading-edge imaging modality diffusion-weighted MRS has recently been shown to be sensitive to an experimental model of neuroinflammation in humans.^98^ Thus, it will be useful for future research to test the associations of these rapidly developing neuroimaging biomarkers with depressive symptoms amongst people living with HIV.

In a scoping review of the field, it is useful to consider which communities and lived experiences are not yet represented in the literature. This review found that the majority (70%) of all eligible studies investigating associations between inflammatory markers and depression in people living with HIV were carried out in the US. There was also a clear skew towards a greater proportion of men in participant samples (Fig. 2). Studies that included a greater proportion of women were conducted in Africa or Asia, with the exception of publications reporting findings from the Women’s Interagency HIV Study in the US, which intentionally recruited only women participants. No studies included participants who were transgender or gender non-conforming, despite the rates of HIV and depression being elevated in these groups.^99,100^ Participants in included studies also tended to be older, with none of the included studies investigating biomarkers of depression in children or adolescents living with HIV. More people live with HIV in the Global South, and the majority of these are women.^101,102^ Biomarkers of depression have been shown to significantly differ based on sex or gender,^103^ with different biomarkers found to be elevated in men with depression compared to women with depression.^104^ Additionally, women living with HIV exhibit greater systemic inflammation compared to men living with HIV.^105^ These demographic factors—age, sex and gender, and geography—interact significantly such that the prevalence of depression is higher in young women in the Global South.^106^ Therefore, the participant samples in included studies do not reflect the global epidemiology of HIV or depression. Effective translation of biomarker discovery into predictive or therapeutic tools will require careful consideration of differences in immune activation by factors such as age, sex, gender, and geography. Equitable cross-border collaborations are necessary to ensure greater inclusion in future research, as recommended elsewhere.^107^

## Conclusions and hypothesized mechanisms

This review aimed to identify studies investigating associations between depression or depressive symptoms and biomarkers of neuroinflammation and related processes in people living with HIV. Eligible studies utilized a variety of depressive symptom scales and assessed 55 biomarkers across neuroinflammation, chemotaxis, systemic inflammation, monocyte activation, immunometabolism, coagulation and neurogenesis. A key limitation of this review was that statistical reporting was highly variable, with many studies not reporting any measures of effect size at all. As a result, we could not conduct a meta-analysis of effect size estimates or assess whether studies in the field showed evidence of publication bias. Few included studies met our validation criteria for a grade of 3, which represented a biomarker which was significantly associated with depression only in people living with HIV (not in controls) and correlated with depressive symptom severity. Given this, for many biomarkers in this review, it is difficult to ascertain whether the significant associations observed in included studies are reliably dependent on HIV status and offer clinical utility. Finally, study screening and data extraction were carried out by a single reviewer, and the search was restricted to studies published in English, which are notable limitations to the methodology of this review.

Regardless of these limitations, this review identified promising evidence that increased concentrations of IL-6, TNF-α, neopterin, IP-10, sCD14, sCD163 and d-dimer may be linked to greater depressive symptoms among people living with HIV. Conversely, higher concentrations of tryptophan and BDNF may be protective, since early evidence indicates significant negative associations between these biomarkers and depressive symptoms in people living with HIV. The biomarkers MCP-1 and IL-8 as well as the Kyn:Trp ratio were assessed in several studies, but no significant associations with depression were observed in the majority of these studies, indicating that these biomarkers may not substantially influence HIV-associated depression. The remaining biomarkers in the included studies were only assessed in a few studies, with little evidence for a significant association with depression in people living with HIV.

These biomarkers are known to interact with each other as part of the (neuro)inflammatory response. For instance, TNF-α regulates the release of chemokines such as MCP-1 and IP-10.^108^ The cytokines TNF-α and IL-1β regulate the production of IL-6, all of which in turn induce the release of CRP.^109^ Soluble monocyte activation markers such as sCD14 can induce the production of these cytokines.^110^ These inflammatory biomarkers may also trigger the kynurenine pathway of tryptophan metabolism, thus contributing to immuno-metabolic dysfunction, and inhibit the production of BDNF, thus impairing neurogenesis and synaptic plasticity. Kynurenine and other immunometabolites may similarly contribute to the release of chemokines such as MCP-1 and IL-8.^111^ Concentrations of MCP-1 and IP-10 are correlated with lower homovanillic acid, a dopamine metabolite, which in turn is correlated with depressive symptoms.^40^ Alterations in inflammatory biomarkers may thus also interact with dopaminergic signalling deficits to produce depressive symptoms.^112^ Together, these biomarkers thus represent feed-forward loops of neuroinflammation and related physiological processes which may drive the pathogenesis of depressive symptoms as a result of CNS HIV infection.

**Figure 5:**
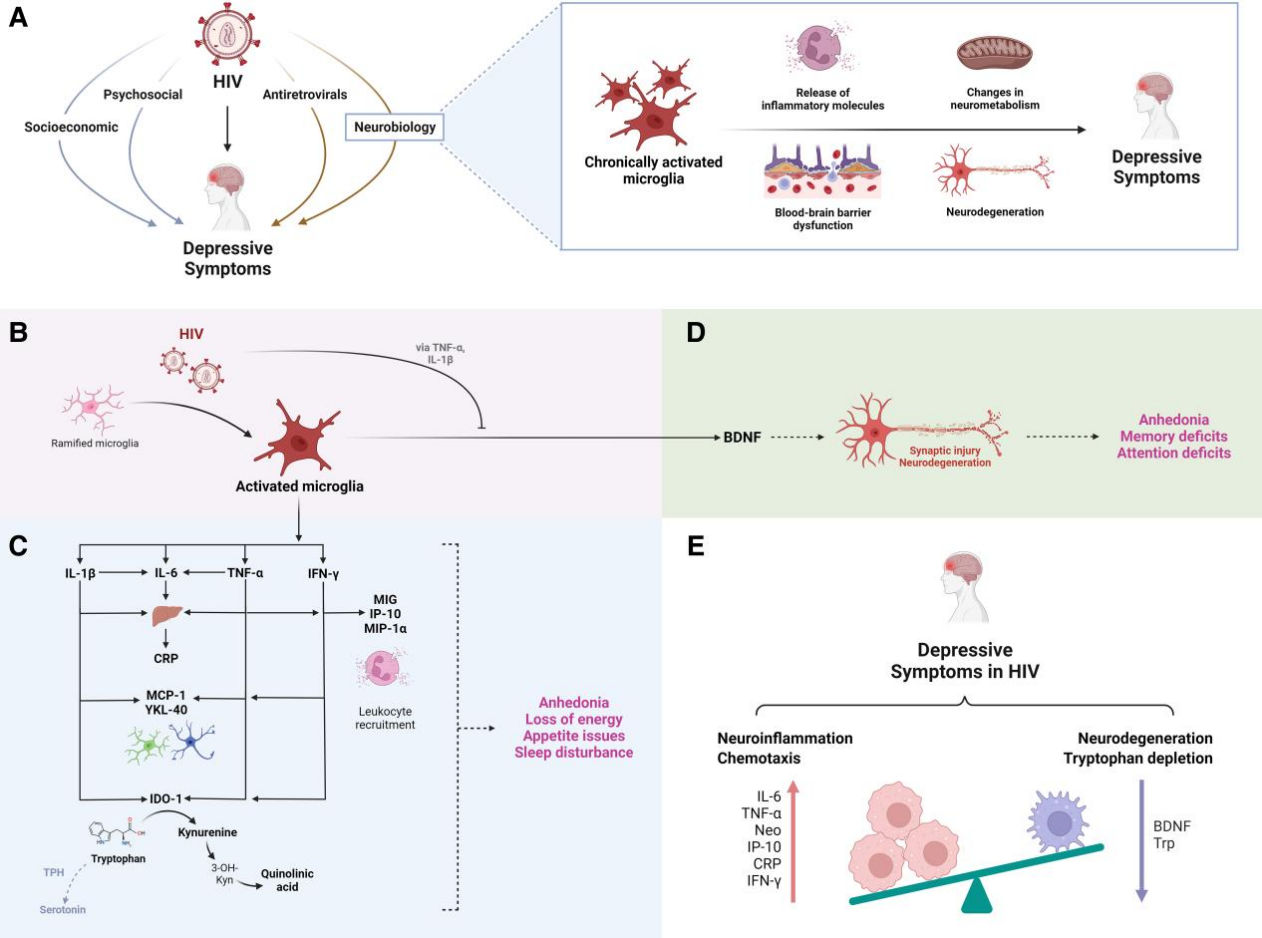
**Visual summary of the hypothesized mechanisms driving the interrelationship between HIV, depression, and neuroinflammation.** (**A**) High rates of depressive symptoms amongst people living with HIV may arise from a syndemic of psychosocial factors (such as social isolation or discrimination), socioeconomic stressors (such as unemployment or food insecurity), side-effects from certain antiretroviral medications, and possible neurobiological disruptions. (**B**) HIV infection in the CNS leads to the activation of resident immune cells, particularly microglia. (**C**) Activated microglia trigger a host of overlapping processes constituting a (neuro)inflammatory response, including the release of various cytokines and chemokines, recruitment of leukocytes, and upregulation of the kynurenine pathway of tryptophan catabolism. This inflammatory response is linked to sickness behaviours, such as loss of energy and sleep or appetite issues, which overlap with depressive symptoms. (**D**) HIV infection also inhibits the production and cellular transport of BDNF via the pro-inflammatory cytokines TNF-α and IL-1β. The inhibition of BDNF in the CNS leads to synaptodendritic injury and neurodegeneration, which in turn contributes to the development of certain depressive symptoms such as memory and attention deficits. (**E**) In summary, HIV may drive the pathogenesis and severity of depressive symptoms by inducing neuroinflammatory cascades, neurodegeneration, and immuno-metabolic dysfunction via chronically activated microglia. A list of abbreviations is available in Supplementary Table 5. Illustration created with BioRender.com.

Our findings support the mechanisms underlying HIV-associated depression which were previously proposed by Del Guerra *et al*.^6^ The evidence identified in this review implicates neuroinflammatory and metabolic biomarkers in HIV-associated depression. Given this, we hypothesize that chronically activated microglia, which trigger these neuroimmunometabolic cascades, are the central link connecting HIV infection in the CNS with the elevated risk for depressive symptoms (Fig. 5). In response to HIV infection, activated microglia stimulates the release of various cytokines and chemokines, which elicit sickness behaviours (such as sleep and appetite issues) associated with depression. HIV infection also leads to the inhibition of the production and cellular transport of BDNF via the pro-inflammatory cytokines TNF-α and IL-1β, as well as increases in blood-brain barrier permeability. The inhibition of BDNF in the CNS leads to synaptodendritic injury and neurodegeneration, which contributes to the development of certain depressive symptoms. Elevations in circulating cytokines such as IL-6 and TNF-α and the upregulation of the kynurenine metabolic pathway, which are induced by HIV infection, are also linked to depressive symptoms. Thus, HIV may drive the pathogenesis and severity of depressive symptoms by inducing neuroinflammatory cascades and immuno-metabolic dysfunction via activated microglia.

In a recent study involving 204 participants with and without HIV, we sought to directly assess potential mediation of the relationship between HIV status and depressive symptoms by inflammatory biomarkers. We showed that statistically adjusting for four biomarkers of inflammation—MIG and TNF-α in plasma and MIP1-α and IL-6 in CSF—attenuated the odds for depressive symptoms in people living with HIV, suggesting that these biomarkers are involved in mediating this risk.^113^ This recent study offers promising evidence that biomarkers of inflammation may be mechanistically involved in the increased risk for depression in people living with HIV and could thus be targeted when developing new or improved therapeutic interventions.

Microglial activation due to inflammatory challenges such as infections is involved in the pathogenesis of depression. Evidence supporting the role of microglia in inflammation and depression has been extensively reviewed elsewhere.^114,115^ Mouse models have shown that inflammatory challenges increased microglial activation in the anterior cingulate cortex (ACC) and induced depressive-like behaviour, whereas mice with deleted ACC microglia did not display depressive-like behaviour in response to experimentally induced inflammation.^116^ Crucially, the microglial activation marker TSPO is elevated in the ACC of people experiencing moderate or severe depressive episodes as well.^117^ Furthermore, novel human brain organoid models with co-cultured microglia have directly demonstrated that microglia release TNF-α and IL-1β in response to HIV infection, which are key cytokines frequently associated with depression.^118^ Taken together, these results strongly suggest that microglia may play a critical role in the pathogenesis of HIV-associated depression.

Although we focus on chronically activated microglia for this discussion, the role of astrocytes in HIV-associated depression also merits investigation. Pre-clinical evidence suggests that astrocytes are important regulators of neuroinflammation and may produce neuroprotective responses to cytokines.^119^ One recent study^73^ specifically explored associations between depressive symptoms and an astrocytic activation marker in people living with HIV. Replication and further investigation of such associations are necessary to determine whether astrocytic markers may be valuable predictive biomarkers or therapeutic targets for HIV-associated depression. Glial cell functions in neuroinflammation do overlap: in an experimental model, both microglia and astrocytes express inflammatory cytokines corresponding to sickness behaviours, but microglial responses precede astrocytic responses.^120^ This temporal distinction may implicate microglia as ‘first responders’ to inflammatory challenges, and therefore, an important target for anti-inflammatory interventions to treat depression.

It is crucial to recognize that neurobiological mechanisms such as those outlined above can only explain in part, if at all, the elevated risk for depression amongst people living with HIV. The pathogenesis of depression in this population is likely driven by multiple contributing factors, including:

– psychosocial and structural factors such as social isolation, racism, or transphobia, which impact many of the communities disproportionately affected by HIV;^121^
– socioeconomic stressors, including unemployment and food or housing insecurity;^122^
– antiretroviral medication, some of which lead to depressive symptoms as a side-effect and may thus confound the measurement of depression in virally suppressed people living with HIV;^52^ or
– managing other co-morbid chronic health conditions unrelated to HIV, such as diabetes.^123^

Tackling the challenge of depression within this population will therefore require concerted efforts to systemically improve quality of life and social support, combat discrimination and stigma, and expand access to psychosocial services alongside biomedical interventions.

## Future directions

This review revealed evidence from the past decade identifying associations of key neuroinflammatory cytokines, chemokines, and monocyte activation markers with depressive symptoms among people living with HIV. Future research may focus on testing the association between biomarkers such as IL-6 and TNF-α in samples that include participants without HIV, in order to establish whether the effects of these biomarkers are reliably influenced by HIV status. Leveraging developing techniques, especially neuroimaging biomarkers, may also support further investigation of neuroinflammation in HIV-associated depression. Although discussion of the gut microbiome was outside the scope of the present review, it must be noted that the composition of the gut microbiome may also modulate the impact of inflammation on depression.^124^ Notably, depression has been linked to gut microbiome dysbiosis in people living with HIV.^125^ These associations may be explored further in future evidence-synthesis exercises.

Future studies must endeavour to fully report test statistics and effect size estimates, not just *P* values, for all biomarkers, including those which do not show statistically significant associations. Reporting effect size estimates such as Odds Ratios (for dichotomous depression/no depression) or correlation coefficients (for continuous depressive symptom scales) will enable a robust meta-analysis of the strength of associations between various biomarkers and depressive symptoms.

Findings from this scoping review may support the translation of existing findings into clinical care for depression in people living with HIV. Biomarkers highlighted in this review may be further investigated in the following ways for appropriate translation:

1. Validating predictive biomarkers, by employing machine learning prediction techniques to large, diverse cohorts of people living with HIV for whom inflammatory biomarkers and depressive symptoms have been measured at multiple time-points.
2. Validating diagnostic biomarkers, by comparing concentrations of biomarkers in large, diverse cohorts of people living with HIV and demographically comparable people without HIV;
3. Validating potential therapeutic interventions, by directly assessing mediating effects of biomarkers (thus identifying biomarkers that are mechanistically involved in the pathogenesis of depression) and developing new therapies or re-purposing existing therapies which target these biomarkers.

Going forward, a strategic balance will be necessary between exploring biomarkers which have shown promise in several studies (as highlighted in this review) and employing broad panels of biomarkers which have not yet been widely assessed. In doing so, future studies can consolidate efforts for biomarker discovery while remaining receptive to serendipitous discoveries. Identifying robust and reliable biomarkers which are correlated with depressive symptoms in people living with HIV may enable the application of immunotherapies to target specific features of depression within this population. These interventions may then be delivered using a precision medicine approach to people living with HIV who exhibit an elevated immune profile alongside depressive symptoms. Together with socioeconomic and psychosocial support interventions, these immunotherapies may thus help reduce the burden of depression among people living with HIV.

## Copyright statement

For the purpose of open access, the author has applied a CC-BY public copyright licence to any Author Accepted Manuscript version arising from this submission.

## Supplementary Material

fcad231_Supplementary_Data

## Data Availability

No primary research data was generated for this article. Systematic search results (initial results, deduplicated results, studies screened using full text, and final included studies) are provided as.CSV files in Supplementary Materials.
