# Supplementary material for: Depression and HIV: a scoping review in search of neuroimmune biomarkers
Source: Brain Commun. 2023 Aug 25;5(5):fcad231. doi: 10.1093/braincomms/fcad231 (PMC10489482; doi:10.1093/braincomms/fcad231)
Supplement: fcad231_Supplementary_Data [file fcad231_supplementary_data.zip › Supplementary_Materials.pdf]

Supplementary Materials

Title of article:
Depression and HIV: a scoping review in search of neuroimmune biomarkers

Author:
Arish Mudra Rakshasa-Loots

Table of Contents

Search strategy	p.1
Supplementary Table 1: Reported effect sizes for the association between inflammatory biomarkers in people living with HIV with (compared to without) depression or depressive symptoms.	p.2
Supplementary Table 2: Cytokines and depressive symptoms in people living with HIV.	p.8
Supplementary Table 3: Other inflammatory biomarkers.	p.12
Supplementary Table 4: Biomarkers of related processes.	p.16
Supplementary Table 5: List of abbreviations.	p.18
References	p.20
Initial systematic search results	**SF2**
Deduplicated search results	**SF3**
Studies screened using full text	**SF4**
Included studies	**SF5**

SF = Supplementary File (provided as separate .CSV files)

Search Strategy

Databases: PubMed, Web of Science

Inclusion Criteria: (1) conducted in a human cohort including people with HIV, (2) report original data, (3) in English, (4) published between January 1, 2013 and November 10, 2022, (5) measure at least one biomarker of inflammation or a related biological process, and (6) test for an association between a biomarker of interest and depression or depressive symptoms

Exclusion Criteria: participants with any other co-infections with HIV, such as tuberculosis or meningitis; studies not reporting original data (reviews, meta-analyses, editorials, and case reports); animal studies; *in vitro* studies; genetic studies

Search Terms: PubMed

(((((HIV OR human immunodeficiency virus OR AIDS OR acquired immunodeficiency syndrome) AND (depression or depressive symptoms)) AND ((inflammation OR neuroinflammation) OR (TSPO OR translocator protein OR Myo-inositol OR Choline OR TNF-α OR Tumor necrosis factor alpha OR IL-1β OR interleukin-1 beta OR IL-6 OR interleukin-6 OR IL-18 OR interleukin-18 OR Neopterin OR CCL2 OR C-C motif chemokine ligand 2 OR MCP-1 OR Monocyte chemoattractant protein-1 OR CCL3 OR C-C motif chemokine ligand 3 OR MIP1-alpha OR Macrophage inflammatory protein-1 alpha OR CCL5 OR C-C motif chemokine ligand 5 OR RANTES OR Regulated upon Activation, Normal T cell Expressed and presumably Secreted OR CXCL9 OR C-X-C Motif Chemokine Ligand 9 OR MIG OR monokine induced by gamma interferon OR CXCL10 OR C-X-C motif chemokine ligand 10 OR IP-10 OR Interferon gamma-induced protein 10 OR Kynurenine OR Quinolinic acid OR BDNF OR brain-derived neurotrophic factor OR NFL OR neurofilament light OR neurofilament light chain OR CRP OR C-reactive protein OR S100A8 OR S100A9 OR YKL-40 OR C3L1 OR chitinase-3 like protein-1)))) AND (((humans[Filter]) AND (2013/1/1:2022/11/10[pdat]) AND (english[Filter])) AND ((humans[Filter]) AND (2013/1/1:2022/11/10[pdat]) AND (english[Filter]))))) NOT ((Review[Publication Type] OR Systematic Review[Publication Type] OR Meta-Analysis[Publication Type] OR Editorial[Publication Type])) Filters: Humans, English, from 2013/1/1 - 2022/11/10

Search Terms: Web of Science

https://www.webofscience.com/wos/woscc/summary/7d43acaa-2f24-49ab-bf7e-e0e62c28ee6c-5d134b4d/relevance/1

HIV OR human immunodeficiency virus OR AIDS OR acquired immunodeficiency syndrome (Topic) AND depression or depressive symptoms (Topic) AND inflammation OR neuroinflammation (Topic) AND TSPO OR translocator protein OR Myo-inositol OR Choline OR TNF-α OR Tumor necrosis factor alpha OR IL-1β OR interleukin-1 beta OR IL-6 OR interleukin-6 OR IL-18 OR interleukin-18 OR Neopterin OR CCL2 OR C-C motif chemokine ligand 2 OR MCP-1 OR Monocyte chemoattractant protein-1 OR CCL3 OR C-C motif chemokine ligand 3 OR MIP1-alpha OR Macrophage inflammatory protein-1 alpha OR CCL5 OR C-C motif chemokine ligand 5 OR RANTES OR Regulated upon Activation, Normal T cell Expressed and presumably Secreted OR CXCL9 OR C-X-C Motif Chemokine Ligand 9 OR MIG OR monokine induced by gamma interferon OR CXCL10 OR C-X-C motif chemokine ligand 10 OR IP-10 OR Interferon gamma-induced protein 10 OR Kynurenine OR Quinolinic acid OR BDNF OR brain-derived neurotrophic factor OR NFL OR neurofilament light OR neurofilament light chain OR CRP OR C-reactive protein OR S100A8 OR S100A9 OR YKL-40 OR C3L1 OR chitinase-3 like protein-1 (Topic) and Article (Document Types) and 2013-01-01 to 2022-11-10 (Publication Date)

Supplementary Table 1: Reported effect sizes for the association between inflammatory biomarkers in people living with HIV with (compared to without) depression or depressive symptoms. Effect sizes were only extracted for biomarkers which were assessed in at least five included studies.

Biomarker	Study	Biofluid	Reported Test	N HIV+	Reported Statistic	Effect Size
	[1]	Plasma	CES-D	493	OR	1.21 (0.85-1.71)
	[2]	Plasma	BDI, CES-D	Test: 22 Validation: 18	Median, IQR Dep vs no dep	N/A
	[3]	Plasma	CES-D	20	Median, IQR Dep vs no dep	-0.56 (-1.01, 0.79) vs -1.01 (-1.01, -1.01)
	[4]	Serum	CES-D-10	143	Pearson correlations	0.20
	[5]	Serum	CES-D-10	160	Pearson correlations	0.24 only as composite
	[6]	Plasma	BDI-II	78	Pearson correlations	0.282
	[7]	CSF	BDI-II	69	N/A	
	[8]	CVL	CES-D	20	Median, IQR Dep vs no dep	0.97 (0.86–1.16) vs 0.58 (0.27–1.00)
**IL-6**	[9]	Plasma	CES-D only as syndemic burden	131	Standardised β	β = -0.02, *t* = -0.53
	[10]	Serum	CES-D	1069	OR only in EIP	1.04 (0.99, 1.10)
	[11]	Serum	MOS-HIV collapsed as dichotomous	407	OR for Q4	0.71 (0.22–2.35)
	[12]	Plasma	MINI Plus	201	aOR for 100/(IL-6+1)	0.979 (0.969; 0.989)
	[13]	N/A	PHQ-9	102	mean (SE) (Class 1 vs 2)	2.32 (0.16) vs 3.43 (0.43)
	[14]	Saliva	SCID-IV and CES-D	Men: 45 Women: 36	Z score rMDD vs noMDD	Women: 0.13 vs 0.37 Men: 0.35 vs -0.12
	[15]	Serum	CES-D	399	Mean log (SD) Dep vs no dep	0.74 (0.70) vs. 0.55 (0.62)
	[16]	Serum	PHQ-9 total	2389	SMD per 1 SD score	0.08
	[17]	Serum	PHQ-9	32	Feature importance	55%

Biomarker	Study	Biofluid	Reported Test	N HIV+	Reported Statistic	Effect Size
TNF-α	[3]	Plasma	CES-D	20	Median, IQR Dep vs no dep	-1.60 (-1.60, -1.60) for both groups
	[4]	Serum	CES-D-10	143	Pearson correlations	0.19
	[5]	Serum	CES-D-10	160	Pearson correlations	0.24 only as composite
	[8]	CVL	CES-D	20	Median, IQR Dep vs no dep	1.47 (1.18–1.52) vs 1.12 (-1.3–1.40)
	[18]	CSF	CIDI	91	N/A	
	[18]	Plasma	CIDI	91	N/A	
	[10]	Serum	CES-D	1069	OR only in EIP	1.04 (0.99, 1.10)
	[12]	Plasma	MINI Plus	201	aOR for 500-999	3.98 (1.29; 12.33)
	[13]	N/A	PHQ-9	102	Mean (SE) for Class 1 vs 2	12.82 (1.30) vs 14.89 (1.72)
	[14]	Saliva	SCID-IV and CES-D	Men: 45 Women: 36	Z score rMDD vs noMDD	Women: 0.10 vs -0.40 Men: 0.38 vs -0.16
	[19]	Plasma	POMS	109	F statistic	1.0 (p = 0.4)
	[17]	Serum	PHQ-9	32	Feature importance	97%
Neopterin	[6]	Plasma	BDI-II	78	N/A	
	[20]	CSF	BDI-II, POMS	65	N/A	
	[20]	Plasma	BDI-II, POMS	65	N/A	
	[21]	Plasma	HADS-D, PHQ-9	123	Mean (SD) Dep vs no dep	2694 (1736) vs 1730 (1327)
	[21]	CSF	HADS-D, PHQ-9	123	Mean (SD) Dep vs no dep & Pearson's r	3401 (1600) vs 1140 (1736) r = 0.29
	[18]	CSF	CIDI	91	N/A	
	[18]	Plasma	CIDI	91	N/A	
	[22]	CSF	BDI-II	123	Pearson r, only as composite	0.18
	[23]	Plasma	CIDI	70	Hedge's g	0.59
	[24]	Urine	BDI-II but combined with PTSD	88	Mean (SE) Clinical symptoms vs no symptoms	358.52 (41.36) vs 207.08 (64.74)

Biomarker	Study	Biofluid	Reported Test	N HIV+	Reported Statistic	Effect Size
IL-1β	[2]	Plasma	BDI, CES-D	Test: 22 Validation: 18	Median, IQR Dep vs no dep	N/A
	[3]	Plasma	CES-D	20	Median, IQR Dep vs no dep	-0.48 (-0.48, -0.48) for both groups
	[8]	CVL	CES-D	20	Median, IQR Dep vs no dep	0.86 (0.31–1.06) vs 0.92 (0.31–1.89)
	[25]	Plasma	PHQ-9	23	N/A	
	[14]	Saliva	SCID-IV and CES-D	Men: 45 Women: 36	Z score rMDD vs noMDD	Women: 0.15 vs -0.18 Men: 0.45 vs -0.28
	[17]	Serum	PHQ-9	32	Feature importance	30%
sTNFR-II	[6]	Plasma	BDI-II	78	N/A	
	[7]	CSF	BDI-II	69	N/A	
	[10]	Serum	CES-D	1069	OR only in EIP	1.09 (1.03, 1.16)
	[11]	Serum	MOS-HIV collapsed as dichotomous	407	OR for Quartile 4	2.61 (1.39–6.66)
	[17]	Serum	PHQ-9	32	Feature importance	62%
MCP-1 / CCL2	[1]	Plasma	CES-D	493	OR	1.00 (0.99 to 1.02]
	[2]	Plasma	BDI, CES-D	Test: 22 Validation: 18	Median, IQR Dep vs no dep	N/A
	[3]	Plasma	CES-D	20	Median, IQR Dep vs no dep	1.80 (0.29, 2.18) vs 1.82 (0.02, 1.88)
	[6]	Plasma	BDI-II	78	N/A	
	[7]	Plasma	BDI-II	69	N/A	
	[7]	CSF	BDI-II	69	N/A	
	[8]	CVL	CES-D	20	Median, IQR Dep vs no dep	2.36 (1.68–2.58) dep 0.02 (0.02–1.94) controls
	[20]	CSF	BDI-II, POMS	65	N/A	
	[10]	Serum	CES-D	1069	OR only in EIP	0.96 (0.88, 1.04)
	[25]	Plasma	PHQ-9	23	N/A	

Biomarker	Study	Biofluid	Reported Test	N HIV+	Reported Statistic	Effect Size
	[14]	Saliva	SCID-IV and CES-D	Men: 45 Women: 36	Z score rMDD vs noMDD	Women: -0.03 vs -0.05 Men: 0.03 vs -0.15
	[22]	CSF	BDI-II	123	Pearson r, only as composite	0.18
**IP-10 / CXCL10**	[2]	Plasma	BDI, CES-D	Test: 22 Validation: 18	Median, IQR Dep vs no dep	10.92 (10.46, 12.36) vs 9.97 (9.23, 11.62)
	[3]	Plasma	CES-D	20	Median, IQR Dep vs no dep	1.96 (1.24, 2.13) vs 2.14 (2.06, 2.26)
	[8]	CVL	CES-D	20	Median, IQR Dep vs no dep	2.72 (2.56–3.06) vs 2.90 (2.41–3.19)
	[20]	CSF	BDI-II, POMS	65	N/A	
	[10]	Serum	CES-D	1069	OR only in EIP	1.09 (1.03, 1.16)
	[25]	Plasma	PHQ-9	23	Spearman correlation	0.351
	[14]	Saliva	SCID-IV and CES-D	Men: 45 Women: 36	Z score rMDD vs noMDD	Women: 0.27 vs 0.20 Men: 0.03 vs -0.45
	[22]	CSF	BDI-II	123	Pearson r	0.22
**IL-8 / CXCL8**	[2]	Plasma	BDI, CES-D	Test: 22 Validation: 18	Median, IQR Dep vs no dep	5.45 (4.83, 5.74) vs 4.83 (4.12, 6.07)
	[3]	Plasma	CES-D	20	Median, IQR Dep vs no dep	0.38 (0.38, 0.38) for both groups
	[7]	Plasma	BDI-II	69	N/A	
	[8]	CVL	CES-D	20	Median, IQR Dep vs no dep	2.33 (1.95–2.93) vs 2.36 (2.15–2.93)
	[10]	Serum	CES-D	1069	OR only in EIP	1.04 (0.99, 1.10)
	[14]	Saliva	SCID-IV and CES-D	Men: 45 Women: 36	Z score rMDD vs noMDD	Women: 0.13 vs 0.37 Men: 0.35 vs -0.12
**CRP**	[1]	Plasma	CES-D	493	OR	1.04 (1.00-1.08)

Biomarker	Study	Biofluid	Reported Test	N HIV+	Reported Statistic	Effect Size
	[4]	Serum	CES-D-10	143	Pearson correlations	-0.02
	[5]	Serum	CES-D-10	160	Pearson correlations	-0.05
	[6]	Plasma	BDI-II	78	Pearson correlations	0.145
	[7]	CSF	BDI-II	69	N/A	
	[11]	Serum	MOS-HIV collapsed as dichotomous	407	OR for Q4	4.37 (1.28–5.88)
	[12]	Plasma	MINI Plus	201	aOR	0.95 (0.78; 1.15)
	[26]	Serum	BDI-I	316	aOR	2.46 (1.34-4.52)
	[14]	Saliva	SCID-IV and CES-D	Men: 45 Women: 36	Z score rMDD vs noMDD	Women: 0.15 vs -0.37 Men: 0.05 vs 0.00
	[27]	Plasma	BDI-II	143	Unstandardised coefficient (SE), interaction with global cognition	– 0.025 (0.010)
	[17]	Serum	PHQ-9	32	Feature importance	31%
**sCD14**	[1]	Plasma	CES-D	493	OR	0.94 (0.62 to 1.44]
	[28]	Plasma	CES-D	110	aOR	0.98 (0.73 to 1.32)
	[6]	Plasma	BDI-II	78	N/A	
	[7]	CSF	BDI-II	69	N/A	
	[10]	Serum	CES-D	1069	OR only in EIP	1.09 (1.03, 1.16)
	[22]	CSF	BDI-II	123	Pearson r, only as composite	0.18
	[16]	Serum	PHQ-9 somatic	2389	SMD per 1 SD score	0.02
**d-dimer**	[6]	Plasma	BDI-II	78	Pearson correlations	0.230
	[7]	CSF	BDI-II	69	N/A	
	[15]	Serum	CES-D	399	N/A	
	[16]	Serum	PHQ-9 somatic	2389	SMD per 1 SD score	0.06
	[17]	Serum	PHQ-9	32	Feature importance	42%
**Kyn:Trp**	[2]	Plasma	BDI, CES-D	Test: 22 Validation: 18	Median, IQR of scaled intensity Dep vs no dep	1.49 (1.05, 1.69) vs 0.96 (0.87, 1.35)

Biomarker	Study	Biofluid	Reported Test	N HIV+	Reported Statistic	Effect Size
	[28]	N/A	CES-D	110	aOR	1.04 (0.37 to 2.90)
	[29]	Plasma	MDI	909	aOR	1.27 (0.76 to 2.13)
	[18]	Plasma	CIDI	91	Unstandardised coefficient (B)	χ^2 = 3.458, β = -1.643
	[18]	CSF	CIDI	91	N/A	
	[30]	Plasma	HSCL-D	504	Mean difference	+0.02 for each 10 nM/μM increase
	[31]	Plasma	BDI	55	OR	2.0 (0.9-4.5)
Trp	[2]	Plasma	BDI, CES-D	Test: 22 Validation: 18	Median, IQR of scaled intensity Dep vs no dep	-0.15 (-0.42, 0.20) vs 0.13 (-0.12, 0.38)
	[18]	CSF	CIDI	91	N/A	
	[18]	Plasma	CIDI	91	N/A	
	[30]	Plasma	HSCL-D	504	Mean difference	−0.07 for each 1000 ng/ml increase
	[31]	Plasma	BDI	55	OR	0.5 (0.2-1.4)
	[32]	Plasma	DASS-42	211	Estimate (unclear)	-0.23
Kyn	[2]	Plasma	BDI, CES-D	Test: 22 Validation: 18	Median, IQR of scaled intensity Dep vs no dep	0.36 (0.05, 0.62) vs 0.23 (-0.17, 0.48)
	[29]	Plasma	MDI	909	aOR	1.25 (0.75 to 2.08)
	[18]	CSF	CIDI	91	N/A	
	[18]	Plasma	CIDI	91	N/A	
	[31]	Plasma	BDI	55	OR	1.3 (0.5-3.0)

aOR: adjusted Odds Ratio; BDI: Beck Depression Inventory; CES-D: Center for Epidemiologic Studies Depression Scale; CIDI: Composite International Diagnostic Interview; DASS: Depression Anxiety Stress Scales; "Dep": depression; EIP: exploratory factor analysis-identified inflammatory process; HSCL-D: Hopkins Symptom Checklist-Depression; IQR: interquartile range; MDD: major depressive disorder; MDI: Major Depression Inventory; MINI-Plus: Mini-International Neuropsychiatric Interview; MOS-HIV: Medical Outcomes Study HIV Health Survey; N/A: data not reported; "no dep": no depression; OR: Odds Ratio; PHQ: Patient Health Questionnaire; POMS: Profile of Mood States; PTSD: post-traumatic stress disorder; rMDD: remitted major depressive disorder; SCID-IV: Structured Clinical Interview for DSM-IV; SD: standard deviation; SE: standard error; SMD: standardised mean difference;

Supplementary Table 2: Cytokines and depressive symptoms in people living with HIV. Summary of recent studies investigating neuroinflammatory cytokines in HIV-associated depression.

Biomarker	Study	Biofluid	Association with depression	Association only in people with HIV	Correlation with depressive symptoms	Grade
IL-6	Anderson et al., 2022 [1]	Plasma	N			0
	Cassol et al., 2015 [33]	Plasma	N			0
	Daniels et al., 2022 [3]	Plasma	N			0
	Derry et al., 2022 [4]	Serum	Y	N		1
	Derry-Vick et al., 2022 [5]	Serum	Y	N		1
	Ellis et al., 2020 [6]	Plasma	Y	N		1
	Ellis et al., 2021 [7]	CSF	N			0
	Ghosh et al., 2018 [8]	CVL	N			0
	Jones et al., 2020 [9]	Plasma	N			0
	Lu et al., 2019 [10]	Serum	N			0
	Memiah et al., 2021 [11]	Serum	N			0
	Musinguzi et al., 2018 [12]	Plasma	Y	N		1
	Norcini Pala et al., 2016 [13]	N/A	Y	N		1
	Rubin et al., 2020 [14]	Saliva	Y	N		1
	Saylor et al., 2019 [15]	Serum	Y	N		1
	Stewart et al., 2020 [16]	Serum	N			0
	Zuñiga et al., 2020 [17]	Serum	N			0
TNF-α	Daniels et al., 2022 [3]	Plasma	N			0
	Derry et al., 2022 [4]	Serum	Y	N		1
	Derry-Vick et al., 2022 [5]	Serum	Y	N		1
	Ghosh et al., 2018 [8]	CVL	N			0

Biomarker	Study	Biofluid	Association with depression	Association only in people with HIV	Correlation with depressive symptoms	Grade
	Keegan et al., 2016 [18]	CSF	N			0
	Keegan et al., 2016 [18]	Plasma	N			0
	Lu et al., 2019 [10]	Serum	N			0
	Musinguzi et al., 2018 [12]	Plasma	Y	N		1
	Norcini Pala et al. 2016 [13]	N/A	N			0
	Rubin et al., 2020 [14]	Saliva	Y	N		1
	Woods et al., 2021 [19]	Plasma	N			0
	Zuñiga et al., 2020 [17]	Serum	Y	N		1
Neopterin	Ellis et al., 2020 [6]	Plasma	N			0
	Gold et al., 2014 [20]	CSF	N			0
	Gold et al., 2014 [20]	Plasma	N			0
	Hellmuth et al., 2017 [21]	Plasma	Y	N		1
	Hellmuth et al., 2017 [21]	CSF	Y	N		1
	Keegan et al., 2016 [18]	CSF	N			0
	Keegan et al., 2016 [18]	Plasma	N			0
	Saloner et al., 2020 [22]	CSF	N			0
	Saloner et al., 2022 [23]	Plasma	Y	Y	Y	3
	Williams et al., 2013 [24]	Urine	Y	N		1
IL-1β	Cassol et al., 2015 [33]	Plasma	N			0
	Daniels et al., 2022 [3]	Plasma	N			0
	Ghosh et al., 2018 [8]	CVL	N			0
	Rivera-Rivera et al., 2014 [25]	Plasma	N			0
	Rubin et al., 2020 [14]	Saliva	Y	N		1

Biomarker	Study	Biofluid	Association with depression	Association only in people with HIV	Correlation with depressive symptoms	Grade
	Zuñiga et al., 2020 [17]	Serum	N			0
sTNFR-II	Ellis et al., 2020 [6]	Plasma	N			0
	Ellis et al., 2021 [7]	CSF	N			0
	Lu et al., 2019 [10]	Serum	Y	Y	Y	3
	Memiah et al., 2021 [11]	Serum	Y	N		1
	Zuñiga et al., 2020 [17]	Serum	N			0
IFN-γ	Cassol et al., 2015 [33]	Plasma	N			0
	Derry et al., 2022 [4]	Serum	Y	N		1
	Derry-Vick et al., 2022 [5]	Serum	Y	N		1
	Rivera-Rivera et al., 2014 [25]	Plasma	N			0
IL-1α	Daniels et al., 2022 [3]	Plasma	N			0
	Ghosh et al., 2018 [8]	CVL	N			0
	Rivera-Rivera et al., 2014 [25]	Plasma	N			0
IL-12	Cassol et al., 2015 [33]	Plasma	N			0
	Rivera-Rivera et al., 2014 [25]	Plasma	Y	N		1
sIL6R	Lu et al., 2019 [10]	Serum	Y	Y	Y	3
	Zuñiga et al., 2020 [17]	Serum	N			0
sTNFR-I	Memiah et al., 2021 [11]	Serum	N			0
	Zuñiga et al., 2020 [17]	Serum	N			0
TGF-β	Daniels et al., 2022 [3]	Plasma	N			0
	Ghosh et al., 2018 [8]	CVL	N			0
CathepsinB	Daniels et al., 2022 [3]	Plasma	N			0

Biomarker	Study	Biofluid	Association with depression	Association only in people with HIV	Correlation with depressive symptoms	Grade
G-CSF	Rivera-Rivera et al., 2014 [25]	Plasma	Y	N		1
IFN-α	Cassol et al., 2015 [33]	Plasma	N			0
IL-10	Cassol et al., 2015 [33]	Plasma	N			0
IL-15	Rivera-Rivera et al., 2014 [25]	Plasma	Y	N		1
IL-18	Memiah et al., 2021 [11]	Serum	Y	N		1
MMP9	Rubin et al., 2020 [14]	Saliva	N			0
sGP130	Lu et al., 2019 [10]	Serum	Y	Y	Y	3
sIL1RII	Zuñiga et al., 2020 [17]	Serum	N			0
sIL2Rα	Lu et al., 2019 [10]	Serum	Y	Y	Y	3

Studies are graded against the validation criteria described in **Table 1**. Y = Yes; N = No.

CSF: cerebrospinal fluid; CVL: cervico-vaginal lavage; G-CSF: granulocyte colony-stimulating factor; IFN-α: interferon alpha; IFN-γ: interferon gamma; IL-6: interleukin-6; IL-10: interleukin-10; IL-12: interleukin-12; IL-15: interleukin-15; IL-18: interleukin-18; IL-1α: interleukin-1 alpha; IL-1β: interleukin-1 beta; MMP9: matrix metallopeptidase 9; sGP130: soluble glycoprotein 130; sIL1RII: soluble interleukin-1 receptor II; sIL2Rα: soluble interleukin-2 receptor alpha; sIL6R: soluble interleukin-6 receptor; sTNFR-I: soluble tumor necrosis factor receptor I; sTNFR-II: soluble tumor necrosis factor receptor II; TGF-β: transforming growth factor beta; TNF-α: tumor necrosis factor alpha.

Supplementary Table 3: Other inflammatory biomarkers. Summary of recent studies investigating biomarkers of inflammatory processes in HIV-associated depression.

Biomarker	Study	Biofluid	Association with depression	Association only in people with HIV	Correlation with depressive symptoms	Grade
Chemokines						
MCP-1 / CCL2	Anderson et al., 2022 [1]	Plasma	N			0
	Cassol et al., 2015 [33]	Plasma	N			0
	Daniels et al., 2022 [3]	Plasma	N			0
	Ellis et al., 2020 [6]	Plasma	N			0
	Ellis et al., 2021 [7]	Plasma	N			0
	Ellis et al., 2021 [7]	CSF	N			0
	Ghosh et al., 2018 [8]	CVL	N			0
	Gold et al., 2014 [20]	CSF	N			0
	Lu et al., 2019 [10]	Serum	N			0
	Rivera-Rivera et al., 2014 [25]	Plasma	N			0
	Rubin et al., 2020 [14]	Saliva	N			0
	Saloner et al., 2020 [22]	CSF	N			0
IP-10 / CXCL10	Cassol et al., 2015 [33]	Plasma	Y	Y	N	2
	Daniels et al., 2022 [3]	Plasma	N			0
	Ghosh et al., 2018 [8]	CVL	N			0
	Gold et al., 2014 [20]	CSF	N			0
	Lu et al., 2019 [10]	Serum	Y	Y	Y	3
	Rivera-Rivera et al., 2014 [25]	Plasma	Y	N		1
	Rubin et al., 2020 [14]	Saliva	Y	N		1
	Saloner et al., 2020 [22]	CSF	N			0

Biomarker	Study	Biofluid	Association with depression	Association only in people with HIV	Correlation with depressive symptoms	Grade
IL-8 / CXCL8	Cassol et al., 2015 [33]	Plasma	N			0
	Daniels et al., 2022 [3]	Plasma	N			0
	Ellis et al., 2021 [7]	Plasma	N			0
	Ghosh et al., 2018 [8]	CVL	N			0
	Lu et al., 2019 [10]	Serum	N			0
	Rubin et al., 2020 [14]	Saliva	Y	N		1
MIG / CXCL9	Cassol et al., 2015 [33]	Plasma	N			0
	Rubin et al., 2020 [14]	Saliva	N			0
MIP-1β	Lu et al., 2019 [10]	Serum	N			0
	Rivera-Rivera et al., 2014 [25]	Plasma	N			0
MIP-3α	Daniels et al., 2022 [3]	Plasma	N			0
	Ghosh et al., 2018 [8]	CVL	N			0
Eotaxin	Lu et al., 2019 [10]	Serum	N			0
MCP-4 / CCL13	Lu et al., 2019 [10]	Serum	N			0
MIP-1α	Rivera-Rivera et al., 2014 [25]	Plasma	N			0
Systemic Inflammation Markers						
CRP	Anderson et al., 2022 [1]	Plasma	N			0
	Derry et al., 2022 [4]	Serum	N			0
	Derry-Vick et al., 2022 [5]	Serum	N			0
	Ellis et al., 2020 [6]	Plasma	N			0
	Ellis et al., 2021 [7]	CSF	N			0
	Memiah et al., 2021 [11]	Serum	Y	N		1
	Musinguzi et al., 2018 [12]	Plasma	N			0

Biomarker	Study	Biofluid	Association with depression	Association only in people with HIV	Correlation with depressive symptoms	Grade
	Poudel-Tandukar et al., 2014 [26]	Serum	Y	N		1
	Rubin et al., 2020 [14]	Saliva	Y	N		1
	Saloner et al., 2021 [27]	Plasma	Y	N		1
	Zuñiga et al., 2020 [17]	Serum	N			0
Albumin	Gold et al., 2014 [20]	CSF	N			0
	Gold et al., 2014 [20]	Blood	N			0
	Poudel-Tandukar et al., 2017 [34]	Serum	Y	N		1
BAFF	Lu et al., 2019 [10]	Serum	Y	Y	Y	3
GlycA	Anderson et al., 2022 [1]	Plasma	N			0
uPAR	Ellis et al., 2021 [7]	CSF	N			0
VEGF	Ellis et al., 2021 [7]	Plasma	N			0
			Monocyte Activation Markers			
sCD14	Anderson et al., 2022 [1]	Plasma	N			0
	Chahine et al., 2021 [28]	Plasma	N			0
	Ellis et al., 2020 [6]	Plasma	N			0
	Ellis et al., 2021 [7]	CSF	N			0
	Lu et al., 2019 [10]	Serum	Y	Y	Y	3
	Saloner et al., 2020 [22]	CSF	N			0
	Stewart et al., 2020 [16]	Serum	Y	Y	Y	3
sCD163	Anderson et al., 2022 [1]	Plasma	Y	Y	N	2
sCD27	Lu et al., 2019 [10]	Serum	Y	Y	Y	3
sCD40L	Ellis et al., 2020 [6]	Plasma	N			0

Studies are graded against the validation criteria described in **Table 1**. Y = Yes; N = No.

Biomarker	Study	Biofluid	Association with depression	Association only in people with HIV	Correlation with depressive symptoms	Grade

BAFF: B cell activating factor; CRP: C reactive protein; CSF: cerebrospinal fluid; CVL: cervico-vaginal lavage; G-CSF: granulocyte colony-stimulating factor; GlycA: glycoprotein acetylation marker; IL-8: interleukin-8; IP-10: interferon gamma-induced protein; MCP-1: monocyte chemoattractant protein-1; MCP-4: monocyte chemoattractant protein-4; MIG: monokine induced by gamma interferon; MIP-1β: macrophage inflammatory protein-1 beta; MIP-1α: macrophage inflammatory protein-1 alpha; MIP-3α: macrophage inflammatory protein-3 alpha; sCD14: soluble cluster of differentiation 14; sCD163: soluble cluster of differentiation 163; sCD27: soluble cluster of differentiation 27; sCD40L: soluble cluster of differentiation 40 ligand; uPAR: urokinase plasminogen activator surface receptor; VEGF: vascular endothelial growth factor.

Supplementary Table 4: Biomarkers of related processes. Summary of recent studies investigating biomarkers of processes related to inflammation in HIV-associated depression.

Biomarker	Study	Biofluid	Association with depression	Association only in people with HIV	Correlation with depressive symptoms	Grade
			Coagulation			
d-dimer	Ellis et al., 2020 [6]	Plasma	Y	N		1
	Ellis et al., 2021 [7]	CSF	N			0
	Saylor et al., 2019 [15]	Serum	N			0
	Stewart et al., 2020 [16]	Serum	Y	Y	Y	3
	Zuñiga et al., 2020 [17]	Serum	N			0
			Neurogenesis			
BDNF	Woods et al., 2021 [19]	Plasma	Y	Y	Y	3
			Immunometabolism			
Kyn:Trp	Cassol et al., 2015 [33]	Plasma	N			0
	Chahine et al., 2021 [28]	N/A	N			0
	Drivsholm et al., 2021 [35]	Plasma	N			0
	Keegan et al., 2016 [18]	Plasma	N			0
	Keegan et al., 2016 [18]	CSF	N			0
	Martinez et al., 2014 [30]	Plasma	Y	N		1
	Mukerji et al., 2021 [31]	Plasma	N			0
Trp	Cassol et al., 2015 [33]	Plasma	N			0
	Drivsholm et al., 2021 [35]	Plasma	N			0
	Keegan et al., 2016 [18]	CSF	N			0
	Keegan et al., 2016 [18]	Plasma	N			0
	Martinez et al., 2014 [30]	Plasma	Y	N		1
	Mukerji et al., 2021 [31]	Plasma	N			0
	Vadaq et al., 2022 [32]	Plasma	Y	N		1

Biomarker	Study	Biofluid	Association with depression	Association only in people with HIV	Correlation with depressive symptoms	Grade
Kyn	Cassol et al., 2015 [33]	Plasma	N			0
	Drivsholm et al., 2021 [35]	Plasma	N			0
	Keegan et al., 2016 [18]	CSF	N			0
	Keegan et al., 2016 [18]	Plasma	N			0
	Mukerji et al., 2021 [31]	Plasma	N			0
Acylcarnitines Metabolites	Cassol et al., 2015 [33]	Plasma	Y	N		1
	Mukerji et al., 2021 [31]	Plasma	Y	N		1
Tyr	Keegan et al., 2016 [18]	CSF	N			0
	Keegan et al., 2016 [18]	Plasma	N			0
Phe	Keegan et al., 2016 [18]	CSF	N			0
	Keegan et al., 2016 [18]	Plasma	N			0
Phe:Tyr	Keegan et al., 2016 [18]	CSF	N			0
	Keegan et al., 2016 [18]	Plasma	N			0
KynA	Drivsholm et al., 2021 [35]	Plasma	N			0
Monoamine Metabolites	Cassol et al., 2015 [33]	Plasma	Y	N		1
Neuroactive Steroids	Mukerji et al., 2021 [31]	Plasma	Y	N		1
Phe:Trp	Chahine et al., 2021 [28]	N/A	N			0
Quin	Drivsholm et al., 2021 [35]	Plasma	Y	N		1
Quin:KynA	Drivsholm et al., 2021 [35]	Plasma	Y	N		1

Studies are graded against the validation criteria described in **Table 1**. Y = Yes; N = No.

BDNF: brain-derived neurotrophic factor; CSF: cerebrospinal fluid; Kyn: kynurenine; KynA: kynurenic acid; Phe: phenylalanine; Quin: quinolinic acid; Trp: tryptophan; Tyr: tyrosine.

Supplementary Table 5: List of abbreviations.

BAFF	B cell activating factor
BDI	Beck Depression Inventory
BDNF	brain-derived neurotrophic factor
CRP	C reactive protein
CNS	central nervous system
CSF	cerebrospinal fluid
CVL	cervico-vaginal lavage
DW-MRS	diffusion-weighted magnetic resonance spectroscopy
ELISA	enzyme-linked immunosorbent assay
EIP	exploratory factor analysis-identified inflammatory processes
GFAP	glial fibrillary acidic protein
GlycA	glycoprotein acetylation marker
G-CSF	granulocyte colony-stimulating factor
HSCL-D	Hopkins Symptom Checklist for Depression
HIV	human immunodeficiency virus
IRIS	immune reconstitution inflammatory syndrome
IDO-1	indoleamine 2,3-dioxygenase
IFN-α	interferon alpha
IFN-γ	interferon gamma
IP-10	interferon gamma-induced protein
IL-1α	interleukin-1 alpha
IL-1β	interleukin-1 beta
IL-10	interleukin-10
IL-12	interleukin-12
IL-15	interleukin-15
IL-18	interleukin-18
IL-6	interleukin-6
IL-8	interleukin-8
KynA	kynurenic acid
Kyn	kynurenine
MIP-1α	macrophage inflammatory protein-1 alpha
MIP-1β	macrophage inflammatory protein-1 beta
MIP-3α	macrophage inflammatory protein-3 alpha
MRS	magnetic resonance spectroscopy
MMP9	matrix metallopeptidase 9
MCP-1	monocyte chemoattractant protein-1
MCP-4	monocyte chemoattractant protein-4
MIG	monokine induced by gamma interferon
PHQ	Patient Health Questionnaire
Phe	phenylalanine
PET	positron emission tomography

PTSD	post-traumatic stress disorder
Quin	quinolinic acid
SIMOA	single molecular array
sCD14	soluble cluster of differentiation 14
sCD163	soluble cluster of differentiation 163
sCD27	soluble cluster of differentiation 27
sCD40L	soluble cluster of differentiation 40 ligand
sGP130	soluble glycoprotein 130
sIL1RII	soluble interleukin-1 receptor II
sIL2Rα	soluble interleukin-2 receptor alpha
sIL6R	soluble interleukin-6 receptor
sTNFR-I	soluble tumor necrosis factor receptor I
sTNFR-II	soluble tumor necrosis factor receptor II
TGF-β	transforming growth factor beta
TSPO	translocator protein 18 kDa
Trp	tryptophan
TNF-α	tumor necrosis factor alpha
Tyr	tyrosine
uPAR	urokinase plasminogen activator surface receptor
VEGF	vascular endothelial growth factor

References

1. Anderson, A.M., et al., *Higher Soluble CD163 in Blood Is Associated With Significant Depression Symptoms in Men With HIV.* J Acquir Immune Defic Syndr, 2022. **91**(3): p. 325-333.
2. Cassol, E., et al., *Altered Monoamine and Acylcarnitine Metabolites in HIV-Positive and HIV-Negative Subjects With Depression.* J Acquir Immune Defic Syndr, 2015. **69**(1): p. 18-28.
3. Daniels, J., et al., *Lifetime sexual violence exposure in women compromises systemic innate immune mediators associated with HIV pathogenesis: A cross-sectional analysis.* Womens Health, 2022. **18**: p. 11.
4. Derry, H.M., et al., *Links Between Inflammation, Mood, and Physical Function Among Older Adults With HIV.* Journals of Gerontology Series B-Psychological Sciences and Social Sciences, 2022. **77**(1): p. 50-60.
5. Derry-Vick, H.M., et al., *Pain Is Associated With Depressive Symptoms, Inflammation, and Poorer Physical Function in Older Adults With HIV.* Psychosomatic Medicine, 2022. **84**(8): p. 957-965.
6. Ellis, R.J., et al., *Higher levels of plasma inflammation biomarkers are associated with depressed mood and quality of life in aging, virally suppressed men, but not women, with HIV.* Brain, Behavior, & Immunity-Health, 2020. **7**: p. 100121.
7. Ellis, R.J., et al., *Social isolation is linked to inflammation in aging people with HIV and uninfected individuals.* JAIDS Journal of Acquired Immune Deficiency Syndromes, 2021. **86**(5): p. 600-606.
8. Ghosh, M., et al., *Impact of chronic sexual abuse and depression on inflammation and wound healing in the female reproductive tract of HIV-uninfected and HIV-infected women.* PLoS One, 2018. **13**(6): p. e0198412.
9. Jones, D.L., et al., *Syndemic burden and systemic inflammation, HIV health status, and blood pressure among women with unsuppressed HIV viral loads among women living with HIV.* Aids, 2020. **34**(13): p. 1959-1963.
10. Lu, H.D., et al., *Inflammation and Risk of Depression in HIV: Prospective Findings From the Multicenter AIDS Cohort Study.* American Journal of Epidemiology, 2019. **188**(11): p. 1994-2003.
11. Memiah, P., et al., *Mental health symptoms and inflammatory markers among HIV infected patients in Tanzania.* BMC Public Health, 2021. **21**(1): p. 1113.
12. Musinguzi, K., et al., *Association between major depressive disorder and pro-inflammatory cytokines and acute phase proteins among HIV-1 positive patients in Uganda.* BMC Immunol, 2018. **19**(1): p. 1.
13. Norcini Pala, A., et al., *Subtypes of depressive symptoms and inflammatory biomarkers: An exploratory study on a sample of HIV-positive patients.* Brain Behav Immun, 2016. **56**: p. 105-13.
14. Rubin, L.H., et al., *Remitted depression and cognition in HIV: The role of cortisol and inflammation.* Psychoneuroendocrinology, 2020. **114**: p. 8.

15. Saylor, D., et al., *Interleukin-6 is associated with mortality and neuropsychiatric outcomes in antiretroviral-naïve adults in Rakai, Uganda.* J Neurovirol, 2019. **25**(6): p. 735-740.
16. Stewart, J.C., et al., *Associations of Total, Cognitive/Affective, and Somatic Depressive Symptoms and Antidepressant Use With Cardiovascular Disease-Relevant Biomarkers in HIV: Veterans Aging Cohort Study.* Psychosomatic Medicine, 2020. **82**(5): p. 461-470.
17. Zuñiga, J.A., et al., *Biomarkers panels can predict fatigue, depression and pain in persons living with HIV: A pilot study.* Appl Nurs Res, 2020. **52**: p. 151224.
18. Keegan, M.R., et al., *Tryptophan metabolism and its relationship with depression and cognitive impairment among HIV-infected individuals.* International Journal of Tryptophan Research, 2016. **9**: p. IJTR. S36464.
19. Woods, S.P., et al., *Brain-derived neurotrophic factor (BDNF) is associated with depressive symptoms in older adults with HIV disease.* J Neurovirol, 2021. **27**(1): p. 70-79.
20. Gold, J.A., et al., *Longitudinal characterization of depression and mood states beginning in primary HIV infection.* AIDS Behav, 2014. **18**(6): p. 1124-32.
21. Hellmuth, J., et al., *Depression and Anxiety are Common in Acute HIV Infection and Associate with Plasma Immune Activation.* AIDS Behav, 2017. **21**(11): p. 3238-3246.
22. Saloner, R., et al., *Lower CSF homovanillic acid relates to higher burden of neuroinflammation and depression in people with HIV disease.* Brain Behavior and Immunity, 2020. **90**: p. 353-363.
23. Saloner, R., et al., *Neopterin Relates to Lifetime Depression in Older Adults With HIV on Suppressive Antiretroviral Therapy.* J Acquir Immune Defic Syndr, 2022. **89**(4): p. 454-461.
24. Williams, J.K., et al., *A sexual risk and stress reduction intervention designed for HIV-positive bisexual African American men with childhood sexual abuse histories.* Am J Public Health, 2013. **103**(8): p. 1476-84.
25. Rivera-Rivera, Y., et al., *Depression correlates with increased plasma levels of inflammatory cytokines and a dysregulated oxidant/antioxidant balance in HIV-1-infected subjects undergoing antiretroviral therapy.* Journal of clinical & cellular immunology, 2014. **5**(6).
26. Poudel-Tandukar, K., et al., *C-reactive protein and depression in persons with Human Immunodeficiency Virus infection: The Positive Living with HIV (POLH) Study.* Brain Behavior and Immunity, 2014. **42**: p. 89-95.
27. Saloner, R., et al., *Chronically elevated depressive symptoms interact with acute increases in inflammation to predict worse neurocognition among people with HIV.* Journal of Neurovirology, 2021. **27**(1): p. 160-167.
28. Chahine, A., et al., *Blue Monday: Co-occurring Stimulant Use and HIV Persistence Predict Dysregulated Catecholamine Synthesis.* J Acquir Immune Defic Syndr, 2021. **86**(3): p. 353-360.

29. Drivsholm, N., et al., *Alterations in the Kynurenine Pathway of Tryptophan Metabolism Are Associated With Depression in People Living With HIV.* J Acquir Immune Defic Syndr, 2021. **87**(2): p. e177-e181.
30. Martinez, P., et al., *Reversal of the Kynurenine pathway of tryptophan catabolism may improve depression in ART-treated HIV-infected Ugandans.* J Acquir Immune Defic Syndr, 2014. **65**(4): p. 456-62.
31. Mukerji, S.S., et al., *Low Neuroactive Steroids Identifies a Biological Subtype of Depression in Adults with Human Immunodeficiency Virus on Suppressive Antiretroviral Therapy.* J Infect Dis, 2021. **223**(9): p. 1601-1611.
32. Vadaq, N., et al., *Microbiome-Related Indole and Serotonin Metabolites are Linked to Inflammation and Psychiatric Symptoms in People Living with HIV.* International Journal of Tryptophan Research, 2022. **15**: p. 13.
33. Cassol, E., et al., *Altered monoamine and acylcarnitine metabolites in HIV-positive and HIV-negative subjects with depression.* Journal of acquired immune deficiency syndromes (1999), 2015. **69**(1): p. 18.
34. Poudel-Tandukar, K., et al., *Serum albumin levels and depression in people living with Human Immunodeficiency Virus infection: a cross-sectional study.* J Psychosom Res, 2017. **101**: p. 38-43.
35. Drivsholm, N., et al., *Alterations in the Kynurenine Pathway of Tryptophan Metabolism Are Associated With Depression in People Living With HIV.* JAIDS Journal of Acquired Immune Deficiency Syndromes, 2021. **87**(2): p. e177-e181.
